# Specific Tandem Repeats Are Sufficient for Paramutation-Induced Trans-Generational Silencing

**DOI:** 10.1371/journal.pgen.1003773

**Published:** 2013-10-17

**Authors:** Christiane L. Belele, Lyudmila Sidorenko, Maike Stam, Rechien Bader, Mario A. Arteaga-Vazquez, Vicki L. Chandler

**Affiliations:** 1 Department of Plant Sciences, BIO5, University of Arizona, Tucson, Arizona, United States of America; 2 Swammerdam Institute for Life Sciences, University of Amsterdam, Amsterdam, The Netherlands; University of Minnesota, United States of America

## Abstract

Paramutation is a well-studied epigenetic phenomenon in which *trans* communication between two different alleles leads to meiotically heritable transcriptional silencing of one of the alleles. Paramutation at the *b1* locus involves RNA-mediated transcriptional silencing and requires specific tandem repeats that generate siRNAs. This study addressed three important questions: 1) are the tandem repeats sufficient for paramutation, 2) do they need to be in an allelic position to mediate paramutation, and 3) is there an association between the ability to mediate paramutation and repeat DNA methylation levels? Paramutation was achieved using multiple transgenes containing the *b1* tandem repeats, including events with tandem repeats of only one half of the repeat unit (413 bp), demonstrating that these sequences are sufficient for paramutation and an allelic position is not required for the repeats to communicate. Furthermore, the transgenic tandem repeats increased the expression of a reporter gene in maize, demonstrating the repeats contain transcriptional regulatory sequences. Transgene-mediated paramutation required the *mediator of paramutation1* gene, which is necessary for endogenous paramutation, suggesting endogenous and transgene-mediated paramutation both require an RNA-mediated transcriptional silencing pathway. While all tested repeat transgenes produced small interfering RNAs (siRNAs), not all transgenes induced paramutation suggesting that, as with endogenous alleles, siRNA production is not sufficient for paramutation. The repeat transgene-induced silencing was less efficiently transmitted than silencing induced by the repeats of endogenous *b1* alleles, which is always 100% efficient. The variability in the strength of the repeat transgene-induced silencing enabled testing whether the extent of DNA methylation within the repeats correlated with differences in efficiency of paramutation. Transgene-induced paramutation does not require extensive DNA methylation within the transgene. However, increased DNA methylation within the endogenous *b1* repeats after transgene-induced paramutation was associated with stronger silencing of the endogenous allele.

## Author Summary

Paramutation is a fascinating process in which genes communicate to efficiently establish changes in their expression that are stably transmitted to future generations without any changes in DNA sequences. While paramutation was first described in the 1950s and extensively studied through the 1960s, its underlying mechanism remained mysterious for many years. Over the past ten years paramutation at the *b1* locus in maize was shown to require transcribed, non-coding tandem repeats located 100 kb upstream of *b1*. These repeats generate small RNAs, and mutations in multiple genes mediating small RNA silencing at the transcriptional level prevent paramutation. While underlying mechanisms are shared, current models for RNA-mediated transcriptional silencing that are based on experiments with *S. pombe* and Arabidopsis do not explain many aspects of paramutation. In this manuscript we used a transgenic approach to demonstrate that the *b1* non-coding tandem repeats are sufficient to send and respond to the paramutation signals and that this occurs even when the repeats are not at their normal chromosomal location.

## Introduction

Paramutation is a *trans*-interaction between specific alleles or transgenes that leads to a meiotically heritable change in the expression of one of the participating alleles or transgenes. Originally described for the maize (*Zea mays L.*) *r1* (*red1*) [1] and *b1* (*booster1*) [2] genes, paramutation has since been reported for several other genes in plants (see e.g. [3]–[9]). Paramutation-like interactions have also been described in other species, including Drosophila [10], mammals and humans (for review see [11]).

Paramutation at the *b1* locus provides a powerful system for dissecting the underlying mechanism of paramutation (reviewed in [12]). The *b1* gene encodes a transcription factor that activates the purple anthocyanin biosynthesis pathway. Alterations of *b1* expression lead to a visual change in plant pigmentation, and the amount of pigment is a read-out of the *b1* transcription level [3]. The two *b1* alleles that participate in paramutation are *B-I* (*B-Intense*) and *B'*; *B-I* is highly expressed and specifies dark purple pigmentation of the husk, sheath and tassel of the maize plant, while *B'* is expressed at a much lower level and specifies light streaky pigmentation in the same plant tissues as *B-I* [3], [13]. The high expressing *B-I* allele is unstable and can spontaneously change to *B'* at variable frequencies (can be up to 10%; [13]). In contrast, *B'* is very stable and does not change to *B-I* in wild-type genetic backgrounds [13], [14]. Paramutation occurs when *B'* and *B-I* alleles are combined in one nucleus by crossing. The “paramutagenic” *B'* allele turns the “paramutable” *B-I* allele into *B'* at a 100% frequency. The new *B'* allele (*B-I* in the previous generation) is as heritable and paramutagenic as the original *B'* allele [13]. Alleles that do not participate in paramutation are referred to as neutral [14].

Genetic screens in maize have uncovered a number of genes required for paramutation (reviewed in [12], [15], [16]). All but one gene [17] identified to date share homology with genes involved in the RNA-directed transcriptional silencing pathway in *Arabidopsis* [18], strongly indicating a requirement of this pathway for paramutation.

A necessary step towards further dissecting the mechanism of paramutation is knowledge of the key sequences mediating paramutation, the subject of this work. Previous fine structure recombination studies between *B'* or *B-I* and neutral *b1* alleles revealed that paramutation requires a region spanning ∼6 kb located ∼100 kb upstream of the *b1* transcription start site [19], [20]. This region was also required for high *b1* expression. In *B'* and *B-I*, this region contains seven tandem repeats of an 853-bp sequence that is unique to this location within the maize genome. Notably, an allelic series in which alleles differed only by the number of repeats revealed that multiple repeats are required for paramutation. Alleles with seven and five repeats were fully paramutagenic, alleles with three repeats had reduced paramutagenicity, and alleles with a single repeat were neutral to paramutation [19].

The *B'* and *B-I* alleles are epialleles as they have identical DNA sequences [20]. Consistent with epigenetic regulatory mechanisms defining the *B'* and *B-I* states, the hepta-repeats have distinct chromatin structures in *B'* and *B-I* [19], [21]. The epigenetic mark that correlates best with paramutation ability is DNA methylation. The *B'* repeats have extensive DNA methylation, while the *B-I* repeats have low levels of DNA methylation [21]. There are differences between the alleles in histone modifications and the extent of chromosomal looping between the repeats and the *b1* promoter, but these differences correlate mainly with tissue-specific expression, not the heritable silencing associated with paramutation [21], [22].

The *b1* tandem repeats are transcribed [23] and generate siRNAs [24], yet repeat siRNAs are produced even from alleles that do not participate in paramutation, suggesting *b1* siRNAs are not sufficient for paramutation in the tissues analyzed [24]. However, when the repeat sequence is expressed as a hairpin RNA from a transgene, which generates much higher levels of siRNAs than the endogenous alleles, heritable silencing and paramutation can be reconstructed [24]. This contrasts with two other examples of siRNAs generated from hairpin RNA producing transgenes in maize. These siRNAs effectively silenced homologous promoters, yet that silencing was not heritable [24]. Similar studies using hairpin RNAs to silence promoters in Arabidopsis did not report on heritability (e.g. [25]).

We hypothesize that the tandem repeats of the *B-I* and *B'* epialleles have special properties, which confer the ability to establish and heritably transmit the silenced paramutagenic state of *B'*. In this study, we test this hypothesis by asking whether the tandem repeats themselves are sufficient to send and respond to *trans-*acting paramutation signals, using a series of transgenes containing *b1* tandem repeats. Our results are consistent with the above hypothesis. While paramutation was effectively reconstituted, the repeat transgene-induced silencing of *B-I* was less frequent and showed reduced stability in the next generation relative to endogenous paramutation, which occurs 100% of the time and is always stably transmitted.

## Results

### Transgenes carrying *b1* upstream regulatory sequences encompassing the tandem repeats can silence *B-I* from non-allelic genomic locations

**Figure 1:**
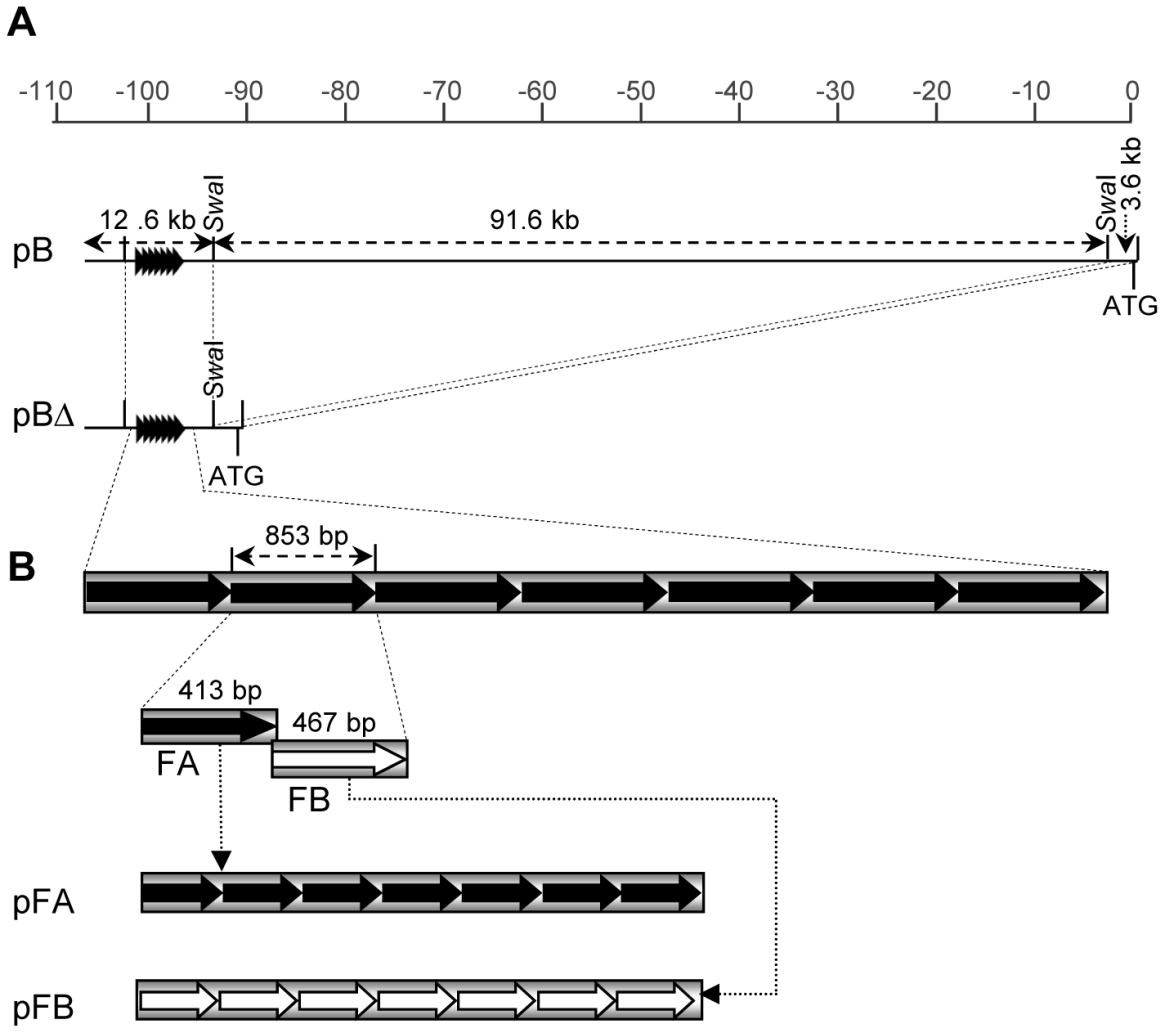
Transgenic constructs used for maize transformation. **A**. Schematic drawing of the upstream *b1* region and the two BAC clones used for plant transformation, pB and pBΔ. The scale at the top shows positions in kilobases (kb) relative to the ATG of the *b1* gene. The 107.8 kb insert in pB contains the *b1* repeats, the first two exons of *b1*, and all the sequences in between. The pBΔ clone is a deletion derivative of the pB clone that lacks 91.6 kb of internal sequences as indicated. The tandem repeats are indicated by arrowheads. **B**. Schematic representation of the pFA and pFB tandem repeat constructs. FA corresponds to one half of the repeat sequence and FB corresponds to the other half. The pFA and pFB tandem repeat constructs contain seven tandem copies of either FA or FB, respectively.

To test whether the *b1* sequences upstream of the transcription start site (TSS) could induce silencing of the *B-I* allele from a non allelic position, two constructs carrying the *b1* repeats and surrounding sequences were used to generate transgenic maize lines: pB, containing the 5′ part of the *b1* transcription unit and 106.2 kb of sequences upstream of the ATG (Figure 1A, [19], [20]) and pBΔ, which had 91.6 kb deleted between the tandem repeats and the proximal promoter of the *b1* transcription unit relative to pB (Figure 1A). These constructs allowed us to also address if, in addition to the tandem repeats, other sequences upstream of the TSS were required for paramutation. For example, the observed transcription of the repeats [23], [24] is likely to be required for paramutation and the promoter sequences driving this transcription might be located outside of the repeats.

**Figure 2:**
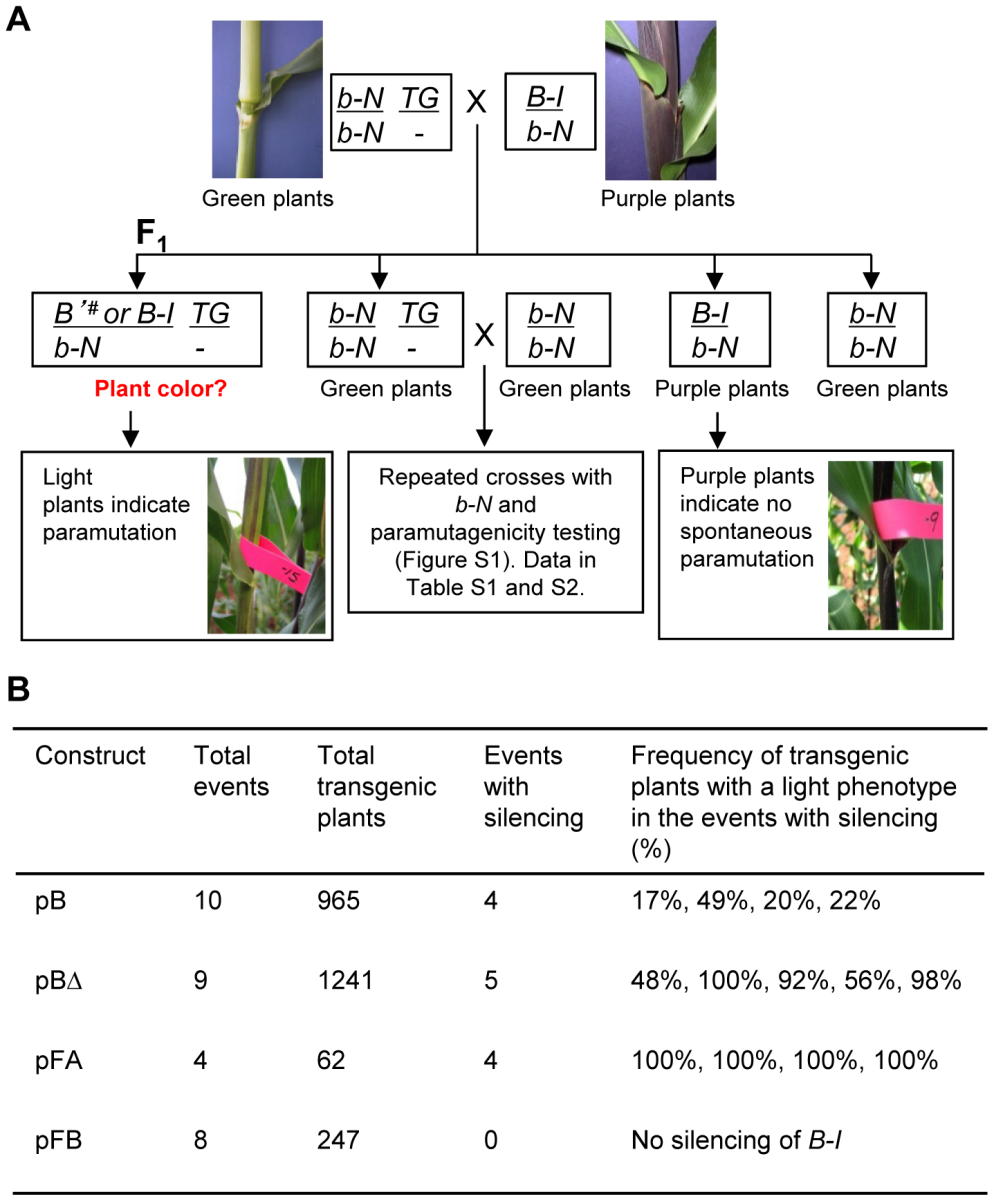
Silencing of the *B-I* allele by transgenes carrying tandem repeat sequences integrated in non-allelic locations. **A**. Crossing strategy for testing the ability of transgenes to induce silencing of the endogenous *B-I* allele. Regenerated transgenic plants, *b-N/b-N*; *TG/-*, were crossed with plants carrying the paramutable *B-I* allele and a neutral *b-N* allele. Silencing of the *B-I* allele was assessed by analyzing the pigmentation of the *B-I/b-N* progeny plants. If *B-I* is silenced by the transgene, indicated by *B'^#^*, transgenic plants should be light. If a transgene is not able to silence *B-I*, all plants, transgenic and non-transgenic, should be dark, unless spontaneous paramutation endogenous of *B-I* to *B'* occurred. Non-transgenic *B-I/b-N* siblings served as controls for spontaneous paramutation of *B-I* to *B'* and should remain dark if spontaneous paramutation does not occur. Any families that showed spontaneous paramutation of *B-I* to *B'* in non-transgenic siblings were removed from further analysis. **B**. Results from the experiments indicated in Panel A for the four constructs diagrammed in Figure 1. Detailed information on each transgenic event is in Table S1 and Table S2. The indicated frequencies of transgenic plants with a light phenotype are a compilation of the data obtained for transgenic loci maintained up to six generations in the presence of a neutral *b1* allele (outlined in Figure S1). Frequency of light plants was calculated by diving the number of light transgenic plants over the total number of transgenic plants. The designation *b-N* is used to represent the neutral alleles used in the crosses; *b-N* alleles carry a single 853 bp repeat unit, do not participate in paramutation and produce no plant pigment.

The *Hi-II* maize stock used for transformation carried recessive neutral *b1* alleles (designated as *b-N*) that do not participate in paramutation and do not confer anthocyanin plant pigment (V. Chandler, unpublished data), enabling the monitoring of silencing activity of the transgenes after crossing the regenerated transgenic plants to *B-I*. To test whether the sequences within either construct could mediate *B-I* silencing, the primary transgenic plants were crossed with plants carrying the paramutable *B-I* allele and a neutral *b-N* allele (Figure 2A). The presence of the neutral allele provided a means to propagate the transgenes in the absence of *B-I* (Figure 2A), which was done for multiple generations by crossing with *b-N* testers (Figure S1). To test the ability of transgenes to induce silencing, transgenic plants at different generations of propagation were crossed with *B-I* (Figure S1, Table S1). Scoring of plant pigment of the *B-I/b-N* progeny carrying transgene loci (*TG/-*) revealed that four out of ten pB, and five out of nine pBΔ transgene loci induced silencing of *B-I* (Figure 2B). In the transgenic events with silencing, the frequencies of silencing varied across multiple generations, ranging from 17 to 100% (Figure 2B, Table S2). The phenotypes of plants showing transgene-induced silencing of *B-I* were very similar to those showing *B'*-induced paramutation of *B-I* (Figure 2A and data not shown). In this paper, the transgene-induced silenced state of *B-I* is noted as *B'^#^* to signify the transgenic origin of this state, in contrast to paramutation induced by the endogenous *B'* allele. Non-transgenic sibling plants (*B-I/b-N*) served as controls for spontaneous paramutation of *B-I* to *B'*, which can happen frequently [14]. Data from families showing spontaneous paramutation in non-transgenic siblings were not included in this paper.

Our results indicate that silencing of *B-I* can be mediated by sequences in ectopic, i.e. non-allelic, locations, paving the way for using a transgenic approach to further dissect the minimal sequences required for paramutation. Furthermore, these results demonstrate that a sub-fragment of the *b1* locus, containing primarily the tandem repeats and the 5′ part of the *b1* transcription unit, is sufficient to establish *B-I* silencing.

### Tandem repeats of a 413 bp sub-fragment of the 853 bp repeat unit are sufficient to induce silencing of *B-I*

The most prominent feature within the 16.3 kb sequence contained in the pBΔ construct are the seven 853 bp tandem repeats, and as paramutation strength correlates with the number of repeats [19], they were strong candidates for the minimal sequences mediating paramutation. To determine which part of the repeat sequence is needed to induce silencing, the 853 bp tandem repeat unit was dissected into two halves based on their different GC content; one half (hereafter referred to as FA) is 48% AT-rich, while the other half (hereafter referred to as FB) is 68% AT-rich [19] (Figure 1B). PCR-amplified sub-fragments (FA or FB halves) were ligated in head-to-tail orientation to form seven tandem repeats (Figure 1B). Constructs carrying the FA and FB hepta-repeats, pFA and pFB, were then transformed into maize and the resulting twelve transgenic events were tested for their ability to induce *B-I* silencing, similar to the approach used for pB and pBΔ transgenic loci (Figure 2A). Results revealed that all four pFA transgenic events induced *B-I* silencing at 100% frequency, indicating the pFA transgene contains all sequences sufficient for *trans*-silencing (Figure 2B, Table S2). None of the eight pFB transgenic events induced *B-I* silencing (Figure 2B, Table S2), suggesting the FB sequences were not sufficient for *trans*-silencing. Because pFB transgenic events do not induce silencing they serve as controls demonstrating that specific repeated sequences mediate silencing of *B-I*.

### The *B'^#^* state is heritable and paramutagenic

One of the defining features of *b1* paramutation is that *B'* is fully paramutagenic to *B-I* and the silencing is heritable [13]. To assay whether the transgene-induced *B'^#^* silenced state was heritable and paramutagenic, plants carrying *B'^#^* alleles, induced by three independent pBΔ and two independent pFA transgenic loci, were crossed with plants heterozygous for the paramutable *B-I* and a neutral *b-N* allele (Figure S2A). Assaying the phenotype of the resulting non-transgenic *B'^#^/b-N* progeny revealed that the silenced *B'^#^* phenotype was heritable in the majority (78–100%) of the non-transgenic plants (Table 1 ^b^). Assaying the *B'^#^/B-I* non-transgenic progeny revealed that the *B'^#^* states were often paramutagenic (41–100%; Table 1 ^d^). To distinguish the various epigenetic states, we use *B'∧* to signify a *B-I* allele silenced by *B'^#^*. Together, our results demonstrate that pBΔ and pFA-induced silencing of *B-I* to *B'^#^* can recapitulate the two key characteristics of paramutation; the silenced *B'^#^* state can be transmitted to progeny and it can be paramutagenic, inducing the *B'∧* silenced state in the absence of the inducing transgene.

**Table 1:** Heritability and paramutagenicity of transgene-induced *B'^#^* silencing^a^.

Transgenic construct	Transgenic event	Heritability of *B'^#^* as determined by phenotype of non-transgenic *B'^#^/b-N* plants				Paramutagenicity of *B'^#^* as determined by phenotype of non-transgenic (*B'∧ or B-I*)/*B'^#^* plants			
		One generation with transgene^b^		Two generations with transgene^c^		One generation with transgene^d^		Two generations with transgene^e^	
		Total Plants	Frequency of light plants	Total Plants	Frequency of light plants	Total Plants	Frequency of light plants	Total plants	Frequency of light plants
pBΔ	3-33	52	84%	92	100%	19	73%	46	100%
	3-39	51	78%	36	100%	24	41%	73	100%
	3-57	85	96%	55	100%	24	91%	84	100%
pFA	60-05	77	100%	Nt	Nt	19	100%	Nt	Nt
	60-11	51	100%	Nt	Nt	14	100%	Nt	Nt

a Crossing scheme to test *B'^#^* heritability and paramutagenicity is shown in Figure S2; the data are summarized in this table.

b Heritability of *B'^#^* after one generation in the presence of the transgene.

c Heritability of *B'^#^* after two generations in the presence of the transgene.

d Paramutagenicity of *B'^#^* after one generation in the presence of the transgene.

e Paramutagenicity of *B'^#^* after two generations in the presence of the transgene.

Nt, not tested because paramutation was already 100% after one generation with the transgene.

Unlike the state induced by the *B'* allele, the heritability and paramutagenicity of the *B'^#^* state was not fully penetrant and the frequency varied between the different pBΔ transgenic events. To test whether prolonged exposure to the pBΔ transgenes would increase the heritability and paramutagenicity of *B'^#^*, *B'^#^/b-N; TG/-* plants carrying *B'^#^* alleles that had been exposed to the transgenes for two subsequent generations were crossed with either *b-N* or *B-I* (Figure S2B). For all three pBΔ transgenic events tested, subsequent generation in the presence of the transgene increased the heritability and paramutagenicity of the *B'^#^* state to 100% (Table 1 ^ce^). This could be because of prolonged *in trans* interactions between the transgene and *B'^#^*. Spontaneous paramutation of *B-I* can, however, not be completely ruled out.

### Exposure to *B'* increases paramutagenicity of pB and pBΔ, but not pFB transgenes

Roughly half of the pB and pBΔ transgenic events, and all of the pFB transgenic events were not paramutagenic (Figure 2B, Table S2). As the endogenous *B'* and *B-I* alleles have identical DNA sequences but differ in chromatin structure, expression levels and paramutation properties [19], [21], one possibility was that the transgenic events that were not paramutagenic might have assumed a *B-I-*like epigenetic state upon integration. If that was true, such transgenes should become paramutagenic upon exposure to *B'*. To test this hypothesis, *b-N/b-N; TG/-* F_1_ plants (as indicated in Figure 2A and Figure S3), which had never been crossed to *B-I*, but whose siblings crossed to *B-I* demonstrated they carried non-paramutagenic or weakly paramutagenic transgenic events, were crossed to *B'*. The resulting transgenic progeny plants were then crossed to *B-I* to determine if the paramutagenicity of the transgenes had increased (crosses described in Figure S3). Results shown in Table 2 demonstrate that four out of seven pB, and three out of four pBΔ transgenic events became highly paramutagenic.

**Table 2:** Ability of non-paramutagenic or weakly paramutagenic events to become more paramutagenic upon exposure to *B'*.

Construct	Transgenic event	Average paramutagenicity^a^	Transgene paramutagenicity after exposure to *B'*		
			Total number *(B'^#^ or B-I)/b-N; TG/-* plants^b^	Frequency of light plants	Summary on paramutagenicity
pB	4-27	49%	179	99%	Increased
	4-43	22%	163	99%	Increased
	4-03	0%	131	92%	Increased
	4-06	0%	131	97%	Increased
	4-12	0%	183	0%	Not changed
	4-14	0%	177	0%	Not changed
	4-23	0%	116	0%	Not changed
pB*Δ*	3-03	48%	139	100%	Increased
	3-46	56%	87	100%	Increased
	3-34	0%	117	100%	Increased
	3-47	0%	110	0%	Not changed
pFB	61-03	0%	16	0%	Not changed
	61-04	0%	70	0%	Not changed
	61-06	0%	11	0%	Not changed
	61-07	0%	76	0%	Not changed
	61-10	0%	65	0%	Not changed
	61-13	0%	32	0%	Not changed
	61-28	0%	49	0%	Not changed

a Frequencies are as indicated in Table S2.

b Crossing scheme used to derive these plants is shown in Figure S3.

One potential explanation for the increased paramutagenicity could be spontaneous paramutation of the transgenic loci instead of an interaction with *B'*. The frequency of spontaneous paramutation of the transgenes can be estimated by carrying the transgenes for multiple generations with only neutral *b1* alleles and then testing their ability to induce paramutation of *B-I* (shown in Figure S1 and Table S1). While there was some variability from generation to generation among the weakly paramutagenic events, none of the weakly paramutagenic transgenes became fully paramutagenic unless crossed to *B'*. For example, with event number 3-46, its paramutation frequency ranged from 36 to 85% over six generations with neutral alleles. In contrast, after one generation with *B'*, its paramutation frequency was 100%. Similarly, several transgenes only became paramutagenic upon crossing with *B'*. For example, event 4-06 was not paramutagenic when carried for four generations with neutral *b-N* alleles (0% paramutagenicity, Table S1), but became highly paramutagenic (97%) after only one generation with *B'* (Table 2). We refer to these transgenic events as paramutable to distinguish them from the paramutagenic transgenes, which did not require crosses with *B'* to become paramutagenic. The ability of certain transgenes to become paramutagenic only after exposure to *B'* suggested that upon integration these transgenes initially assumed a *B-I*-like state. The transgenic events that did not become paramutagenic, even after crossing with *B'*, are referred to as neutral. In contrast to the majority of the pB and pBΔ transgenic events, none of the seven pFB transgenic events tested showed any paramutagenicity after exposure to *B'* (Table 2), suggesting that the repeat sequences in the pFB transgenes were not sufficient to receive and/or heritably transmit the paramutation signal.

### Transgenic events have complex structures and paramutagenicity does not strictly correlate with the number of repeats

**Figure 3:**
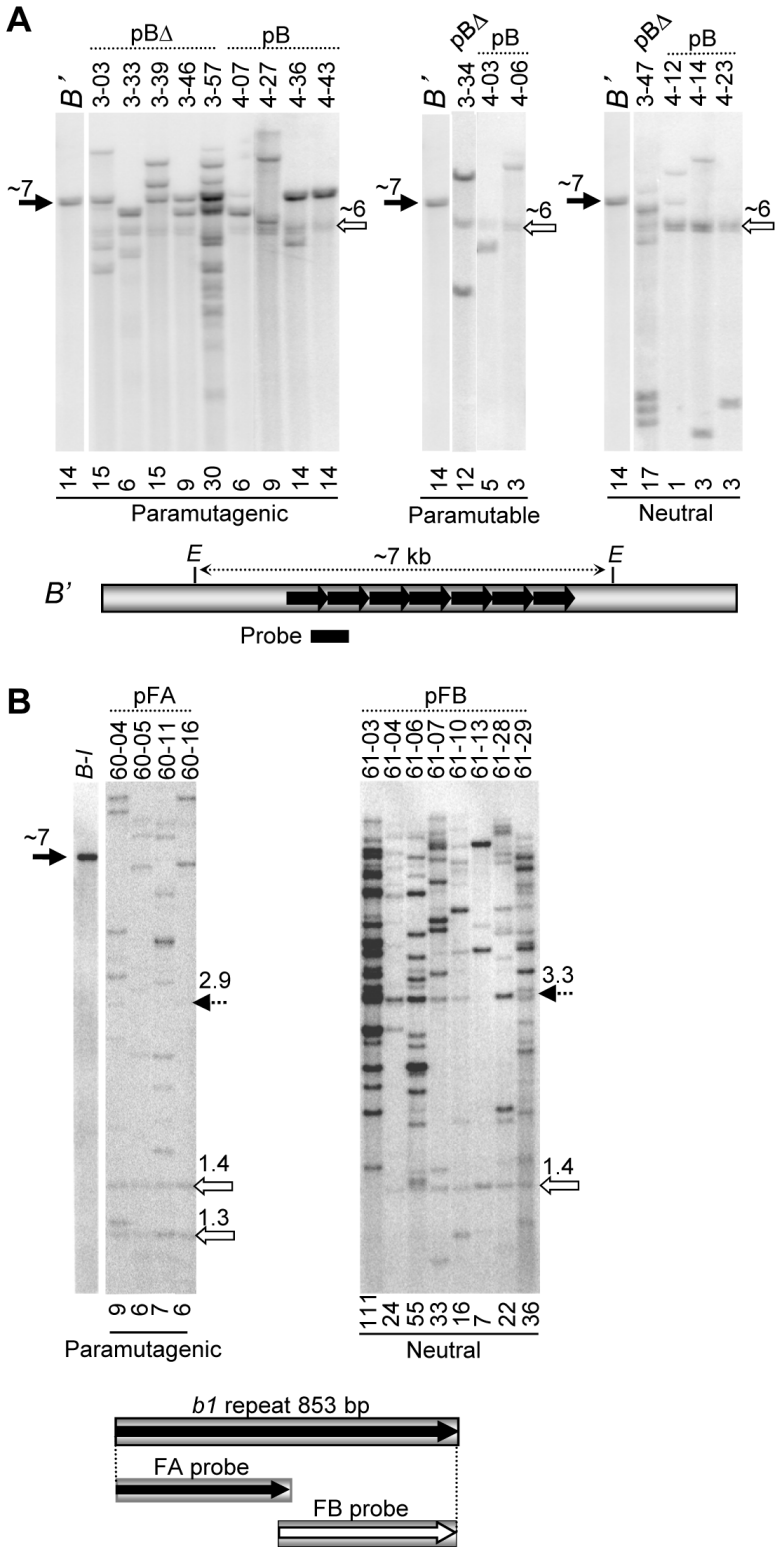
DNA blot analysis of maize transgenic events. The numbers above the arrows indicate approximate fragment sizes in kb. **A**. DNA blot analysis of the paramutagenic, paramutable, and neutral pB and pBΔ transgenic loci. Genomic DNA from transgenic plants was digested with *Eco*RI (E) and blots were hybridized with the tandem repeat probe shown as a bar below the map. The *B'* allele was used as a control to indicate the ∼7 kb *Eco*RI fragment containing the seven tandem repeats (black arrow). All transgenic plants were heterozygous for two different neutral *b1* alleles, each containing a single copy of the repeat unit, together producing a ∼6 kb doublet upon digestion (open arrow). Transgenic event number and construct names are shown above the lanes, while the approximate repeat copy number, estimated using phosphor imaging analysis, is shown below each lane. **B**. DNA blot analyses of plants carrying the pFA or pFB transgenes; genomic DNA was digested with *Bam*HI and *Bgl*II, which cut on either side of the tandem repeat array. The FA and FB fragments diagrammed below the blots were used as probes. Bands corresponding to *B-I* (∼7 kb) and the neutral *b1* alleles (1.4 kb and 1.3 kb) are indicated by black and open arrows, respectively. The 2.9 and 3.3 kb bands corresponding to the intact FA and FB tandem arrays, respectively, are indicated by dotted arrows.

Failure of some transgenic events to participate in paramutation could be attributed to several factors. Transgenes may be truncated and not carry tandem repeats, which are absolutely required for endogenous paramutation [19], or they may have integrated in genomic locations that prevent establishment of silencing. To determine how many events had the intact hepta-repeat fragment and to estimate the number of repeat units present in each event, DNA blot analyses (Materials and Methods) were performed on paramutagenic, paramutable and neutral events (see previous section for definitions). As is typical for biolistic transformation, the DNA blot analysis revealed that the pB and pBΔ transgenic plants contained multiple copies of the transgenes, including complete and truncated fragments (Figure 3A), which segregated as a single locus in each independent event. Six of the paramutagenic transgene loci carried an intact hepta-repeat fragment (Figure 3A, black arrow, 7 kb) and three paramutagenic events did not. None of the paramutable or neutral events carried an intact hepta-repeat. Thus, an intact hepta-repeat fragment was associated with paramutagenicity but was not absolutely necessary for an event to be paramutagenic or paramutable. As all of the insertions are complex we cannot rule out that one or more of the transgenic lines also contain repeats in an inverted orientation, a sequence arrangement known to mediate silencing [25], [26]. We favour our hypothesis that it is the tandem repeats mediating paramutation because it is unambiguous from the fine structure mapping that tandem repeats mediate endogenous paramutation [19] and all the transgenic events with an intact tandem hepta-repeat were paramutagenic.

The number of repeat units present within each event was estimated by normalizing to an endogenous fragment containing a single repeat unit (Materials and Methods). In each functional category, paramutagenic, paramutable or neutral, there are examples of transgenic events that have relatively high or low numbers of repeat units (Figure 3A). All transgenic events, except one neutral event (4–12), carried more than one copy of the 853 bp repeat unit. There was not an absolute correlation between the number of the repeats and paramutation activity in the transgenic events (Figure 3A), although all intact hepta-repeat events were paramutagenic. A similar lack of correlation was observed with the pFA and pFB transgenic events (Figure 3B). The pFA transgenic events, which were all highly paramutagenic, had lower copy numbers (6–9 repeat units) than most of the pFB events (seven out of eight events had 16 or more repeat copies), which showed no paramutation ability. In addition, most of the pFB events had an intact fragment containing seven repeats, while none of the pFA events did (Figure 3B). These results confirm that the pFA sequences are sufficient for paramutation, while the pFB sequences are not.

### Paramutation does not require extensive DNA methylation within the transgene repeats

**Figure 4:**
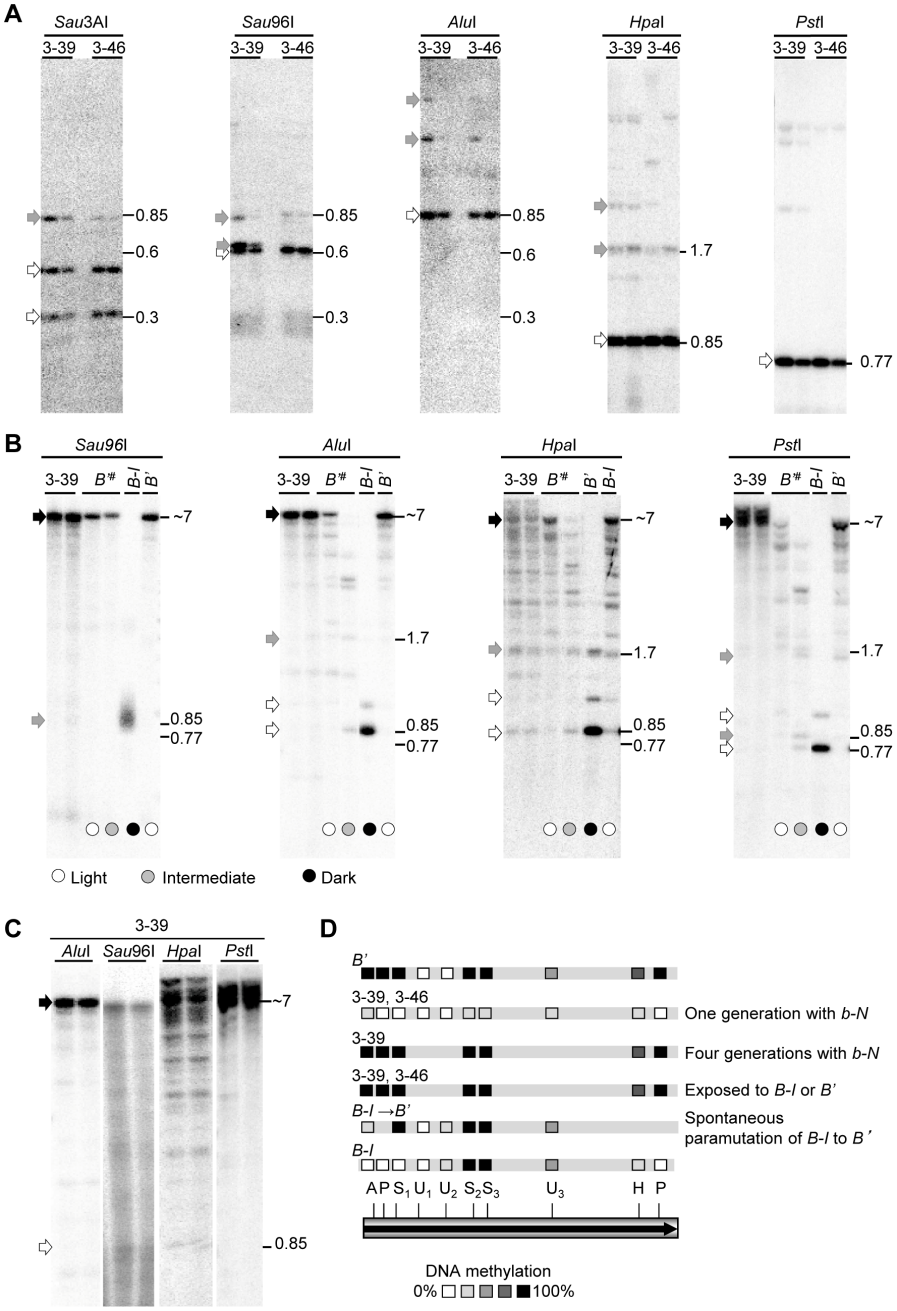
DNA methylation patterns in transgenic and endogenous *b1* repeats. Fragment sizes are indicated by numbers and are in kb. All DNA blots were hybridized with the full *b1* repeat probe (Figure 3A). For all blots, genomic leaf DNA was cut with the methylation insensitive enzyme *Eco*RI to release the ∼7 kb fragment within which DNA methylation was assayed, and with the methylation sensitive enzymes indicated above each blot. Fragments resulting from complete digestion are indicated by open arrows, bands resulting from partial digestion (indicating partial DNA methylation) are indicated by gray arrowheads, while fragments that are the result of no digestion by methylation sensitive enzymes (indicating complete DNA methylation) are indicated by black arrowheads. Representative examples of DNA methylation patterns are shown. **A**. The progeny of two independent paramutagenic pBΔ transgenic events (3-39 and 3-46) were examined. The plants analyzed were the direct progeny of the primary transgenic plants and the transgenes were not yet exposed to *B'* or *B-I*. Results for representative *b-N/b-N; TG/-* plants (Figure 2A) are shown. **B**. *B-I* was exposed to the pBΔ transgenic event 3-39 for one generation, resulting in light pigmented plants, and then the transgene and the newly induced *B'* ^#^ were segregated away from each other. The transgene, *B'* ^#^ and control samples (*B'* and *B-I*) were assayed. Circles at the bottom of the lanes indicate plant pigment phenotypes. **C**. The pBΔ transgenic locus 3-39 was propagated for four generations in a neutral *b-N* background. **D**. Summary of the DNA methylation data for the transgenic 3-39 and 3-46 lines, plants in which *B-I* spontaneously paramutated to *B'* (Figure S4), and the previously determined *B'* and *B-I* patterns [21]. One and four generations with *b-N* indicates the 3-39 allele was propagated for one and four generations in the presence of neutral *b-N* alleles, respectively. The one repeat shown represents all seven repeats. Subscripts indicate specific recognition sites present more than once in each repeat. *Alu*I (A), *Hpa*I (H), *Pst*I (P), *Sau*3AI (U) and *Sau*96I (S).

Relative to *B-I*, the paramutagenic *B'* allele has high levels of cytosine methylation within the tandem repeats [21]. To determine if there was a correlation between the frequency of paramutation and DNA methylation levels at the transgenic repeats, two pBΔ transgenic events, 3-39 and 3-46, were selected for DNA blot analysis. These two events have relatively simple transgene integrations; one intact hepta-repeat fragment and only a few other, truncated repeat-containing fragments (Figure 3A), enabling the interpretation of the DNA blot results. Representative examples of the 3-39 and 3-46 transgenic loci that were in the presence of neutral *b-N* alleles (in the immediate progeny of regenerated transgenic plants) and had not been exposed to *B-I* or *B'*, are shown in Figure 4A. The transgenic repeats were mostly unmethylated within the assayed restriction sites (Figure 4A, open and grey arrows; a total of four 3-39 and seven 3-46 plants were examined). The repeat DNA methylation levels were not only lower than those previously observed for *B'* and for plants undergoing spontaneous paramutation of *B-I* to *B'*, but were also lower than those observed for *B-I* (Figure 4B and 4D; Figure S4) [19], [21]. These results indicate that paramutation can be mediated by transgenic repeats that do not have the DNA methylation levels typical of *B'*.

To test if the methylation levels of the transgene increased after it had mediated paramutation, we examined the 3-39 transgene after it had segregated from the F_1_ between the primary 3-39 transgenic plant and *B-I* [In this F_1_, paramutation occurred at a frequency of 90%, (Table S1)]. The segregating 3-39 repeat transgene was extensively methylated, equivalent to *B'* (Figure 4B; three plants examined). Thus, after paramutation and segregation the transgene was extensively methylated. This could be due to spontaneous increases in DNA methylation or due to interactions between the transgene and the endogenous allele (resulting in paramutation of *B-I* to *B'^#^*), or both. To test for spontaneous DNA methylation within the repeats, we examined the 3-39 transgene maintained in the presence of neutral *b1* alleles for four generations (never exposed to *B-I* or *B'*). We observed a spontaneous increase in the DNA methylation levels in the transgenic repeats (Figure 4C, black arrows, four plants tested) up to the levels observed for the endogenous *B'* repeats (Figure 4B and 4D). Thus, the increased methylation observed within the 3-39 transgenic repeats after encountering *B-I* could be due to spontaneous events.

The 3-46 transgenic event had very low levels of DNA methylation (Figure 4A) in the immediate progeny of the primary transgenic event, and when crossed with *B-I* plants, paramutation occurred at a frequency of 66% (Table S1). After crossing the 3-46 transgene with *B'* and then outcrossing to *B-I*, 100% paramutation was observed. With this one event, we saw that after crossing with *B'*, both the transgene and *B'^#^* had acquired extensive DNA methylation (summarized in Figure 4D and data not shown; a total of six *B' TG/-* plants, and 11 *B'^#^ TG/-* plants were tested). This result indicates that transgenic repeats with low levels of DNA methylation can acquire higher DNA methylation, but more events and individuals need to be examined to determine if increased paramutagenicity correlates with DNA methylation.

A key difference between transgene-mediated and endogenous allele-mediated paramutation is that the resulting silencing of *B-I* to *B'^#^* is less stable when induced by the transgenes than by *B'* (Table 1). To determine whether this difference in silencing, as measured by plant phenotypes, might correlate with the extent of repeat DNA methylation in the endogenous allele, non-transgenic progeny plants segregating *B'^#^* and displaying a range of pigment phenotypes were examined. These individuals derived from outcrossing the *B'^#^*/*b-N*; *TG/-* F_1_ to *b-N* (Figure S2A). Notably, DNA methylation levels within the *B'^#^* repeats, induced by the 3-39 transgene, varied and this variation correlated with the extent of silencing; the more DNA methylation, the lower the plant pigment levels, which are a read-out of the level of *B'^#^* silencing (Figure 4B and data not shown). The same correlation between the extent of silencing and DNA methylation was observed for the 3-46 transgene (data not shown). While the number of individuals examined is small (four 3-39 and six 3-46 plants looked at in total), these data are consistent with a correlation between the level of *B'^#^* silencing and extent of DNA methylation within the endogenous repeats.

### MOP1 is required for transgene-induced *b1* paramutation

Paramutation by the endogenous *B'* allele requires the *Mop1* gene [23], which encodes a protein with high similarity to RDR2, a putative RNA-dependent RNA polymerase required for RNA-directed transcriptional silencing in Arabidopsis [27]. To test whether MOP1 is required for the transgene-induced paramutation, the appropriate crosses were done to assay the ability of three pBΔ transgenes to paramutate *B-I* in the presence of the *mop1-1* mutation (Figure S5). If paramutation was prevented, the segregating progeny should have the *B-I* phenotype, whereas if paramutation occurred, most progeny should have the *B'* phenotype. Analysis of the segregating non-transgenic progeny revealed that the majority of the plants had a *B-I* phenotype (Table 3), indicating that the *mop1-1* mutation prevented the pBΔ transgenes from paramutating *B-I* to *B'^#^*. A few light *B'* plants were observed in three out of twelve testcross families. These could be the result of spontaneous paramutation of *B-I* to *B'*, or because paramutation was not fully prevented in all plants. The observation that MOP1 is required for the transgenes to silence *B-I* demonstrates RNA-mediated mechanisms are involved in transgene-induced paramutation of *B-I*.

**Table 3:** The *mop1-1* mutation prevents pBΔ-induced paramutation of *B-I*.

Transgenic event	Number of dark plants segregating from homozygous *mop1-1* plants in which *B-I* was exposed to the pBΔ transgene^a^			
	Family 1	Family 2	Family 3	Family 4
3-39	12/13 (92%)	5/5 (100%)	7/7 (100%)	1/1 (100%)
3-46	8/8 (100%)	11/18 (61%)	21/26 (80%)	7/7 (100%)
3-57	13/13 (100%)	13/13 (100%)	18/18 (100%)	10/10 (100%)

a Four families, representing the progeny of four individual *mop1-1* homozygotes, were examined for each transgenic event. The families were derived from outcrossing homozygous *mop1-1* plants carrying *B-I* and a paramutagenic *b1* repeat transgene to *b-N* plants (details of the crosses performed are shown in Figure S5). The number of purple plants is shown before the slash, with the total number of plants scored indicated after the slash. The frequency of dark plants is shown in parenthesis.

### *b1* tandem repeats can mediate high expression of a reporter gene

**Figure 5:**
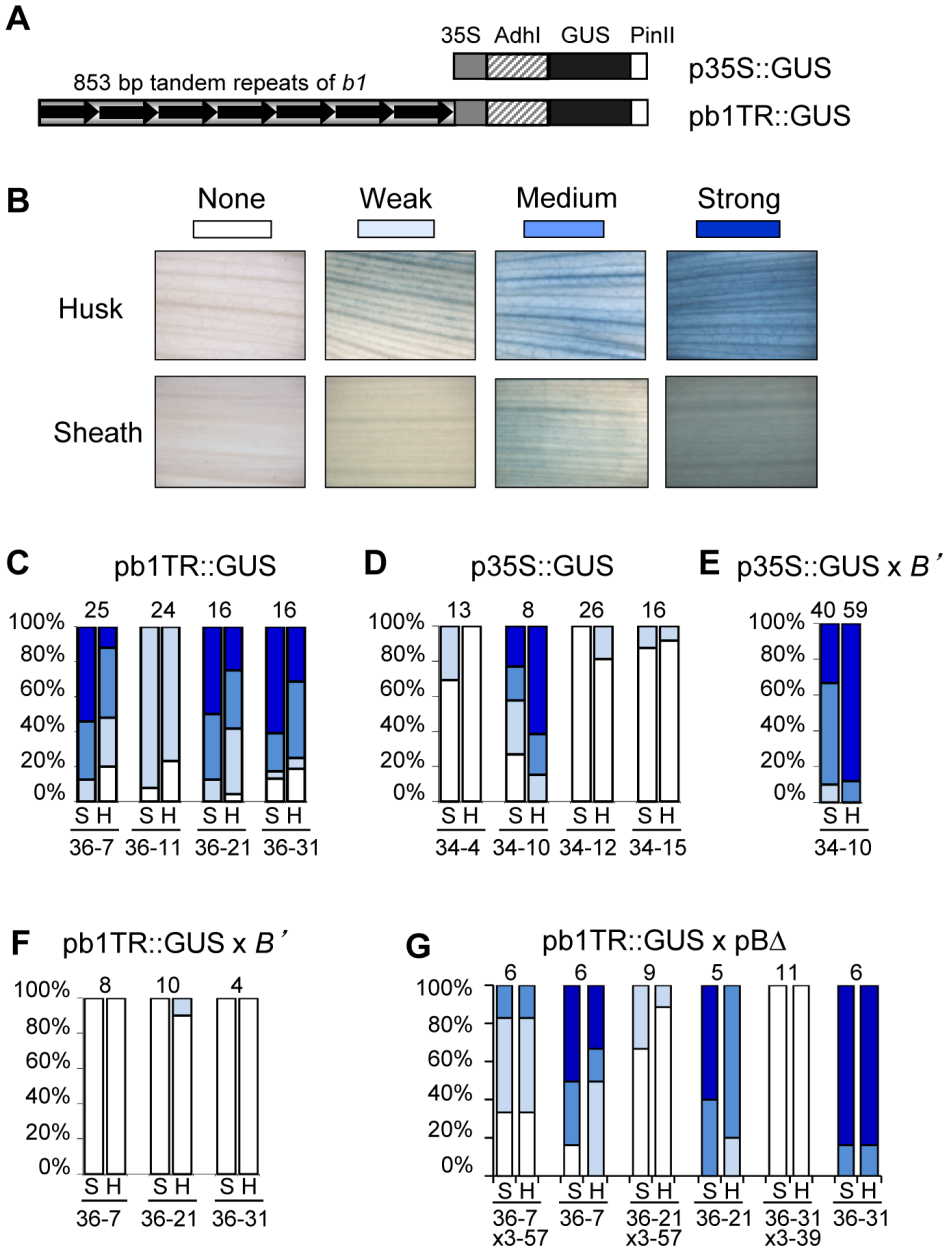
*b1* tandem repeats are sufficient to mediate transcriptional activation in maize. **A**. Drawing of the transgenic constructs used to assay transcriptional regulatory activity of the *b1* repeats. The pb1TR::GUS construct carries the seven tandem *b1* repeats (b1TR). The promoter is the minimal −90 bp Cauliflower Mosaic Virus 35S promoter (35S), which contains no enhancer sequences [28]. GUS is the beta-glucuronidase gene from *E. coli* [29]. *PinII* is the 3′ untranslated region from the potato proteinaseII gene [61]. The p35S::GUS construct, in which GUS expression is driven by the minimal −90 35S promoter, was used as a control. **B**. The scoring scale used to evaluate GUS expression levels in sheath and husk tissues of transgenic plants. Panels **C–G** show the percentage of transgenic plants with specific levels of GUS staining. The shades of blue correspond to the levels of GUS staining indicated in panel B; white signifies no staining. Sheath and husk tissues are denoted as S and H, respectively. The number of plants assayed is shown on the top while the transgenic events are shown below each pair of columns. Panels **C** and **D** show GUS staining levels for pb1TR::GUS and p35S::GUS in a neutral *b1* background. Panels **E** and **F** show GUS staining levels for p35S::GUS and pb1TR::GUS transgene loci in the presence of the paramutagenic *B'* allele. Panel **G** shows GUS staining levels in two classes of progeny derived from crosses between GUS expressing pb1TR::GUS transgenic events and paramutagenic pBΔ events. One class carries both a pb1TR::GUS and pBΔ transgene, the second class carries only a pb1TR::GUS transgene. All transgenic loci shown in panels **C–G** were in a hemizygous state.

In addition to mediating silencing, multiple *b1* tandem repeats are required for high *B-I* expression [19]. It is, however, not known if the repeats are sufficient to mediate high expression or whether additional sequences are needed. To test if the repeats can mediate high expression a construct was produced in which the seven tandem repeats of *B', B-I* (b1TR) were fused to the minimal −90 bp Cauliflower Mosaic Virus 35S promoter (35S) [28] and the GUS (beta-glucuronidase, [29]) reporter gene to generate the pb1TR::GUS transgene (Figure 5A, Materials and Methods). As a negative control, a construct was made that carried only the minimal −90 bp 35S promoter fused to GUS (p35S::GUS). Both constructs were used to generate transgenic maize lines; only lines carrying intact GUS reporter genes were examined for GUS activity (Materials and Methods). Sheath and husk tissues were stained for GUS activity and scored using a graded scale shown in Figure 5B. High GUS activity was observed in pb1TR::GUS events 36-7, 36-21 and 36-31, but not in the event 36-11 (Figure 5C). Southern blot analysis (not shown) revealed that the GUS transgenes in events showing high GUS activity carried about ∼7 repeats (36-7, 1 transgene copy), 6 and 1.5 repeats (36-21, 2 copies), and 4 and 3 repeats (36-31, 2 copies), while the transgenes in the event showing weak GUS activity carried about 3.5 and 2.5 repeats (35-11, 2 copies). Three p35S::GUS control events that contained no repeats showed low GUS activity, while one had high GUS activity (34-10, Figure 5D).

The high GUS activity in the p35S::GUS event 34-10 was unexpected and was hypothesized to be caused by integration of the transgene near an endogenous transcriptional regulatory element. If this hypothesis was correct, the expectation was that the GUS activity should not be silenced by *B'*. In contrast, if the high expression in the pb1TR::GUS events 36-7, 36-21 and 36-31 was mediated by the repeats, *B'* should silence that expression. To test these hypotheses, the p35S::GUS event (34-10) and the three pb1TR::GUS transgenic events strongly expressing GUS (36-7, 36-21 and 36-31) were crossed with the paramutagenic *B'* allele. The three pb1TR::GUS transgenic events were also crossed with two highly paramutagenic pBΔ transgenic events. Results shown in Figure 5E revealed that the expression of p35S::GUS event 34-10 was not affected by *B'*, consistent with the hypothesis that its high expression is caused by integration near an endogenous regulatory element that is insensitive to *B'*. In contrast, all three pb1TR::GUS transgenic events exhibited a significant reduction in GUS activity after exposure to *B'* (Figure 5F) or the paramutagenic pBΔ transgenes (Figure 5G), providing additional support that the high expression was not simply due to insertion next to an endogenous enhancer. The silencing of the pb1TR::GUS transgenic loci in the presence of the paramutagenic pBΔ transgenes was not due to spontaneous paramutation, because for all three pb1TR::GUS loci control transgenic siblings segregating only the pb1TR::GUS transgenes showed higher GUS activity (Figure 5G). Together, these data suggest that the *b1* tandem repeats are sufficient to trigger expression of a heterologous gene and that this expression is sensitive to paramutation.

To determine if the transcriptional regulatory activity within the repeats could be further delineated, transgenic lines containing seven FA or seven FB tandem repeats fused to the minimal p35S::GUS reporter gene were generated (Figure S6). GUS expression was observed in all the four intact pFA::GUS events and the one intact pFB::GUS event. However, because there was only one intact pFB::GUS event available, more experiments will be required to delineate where the transcriptional regulatory activity maps.

### Repeat transgenes produce siRNAs

**Figure 6:**
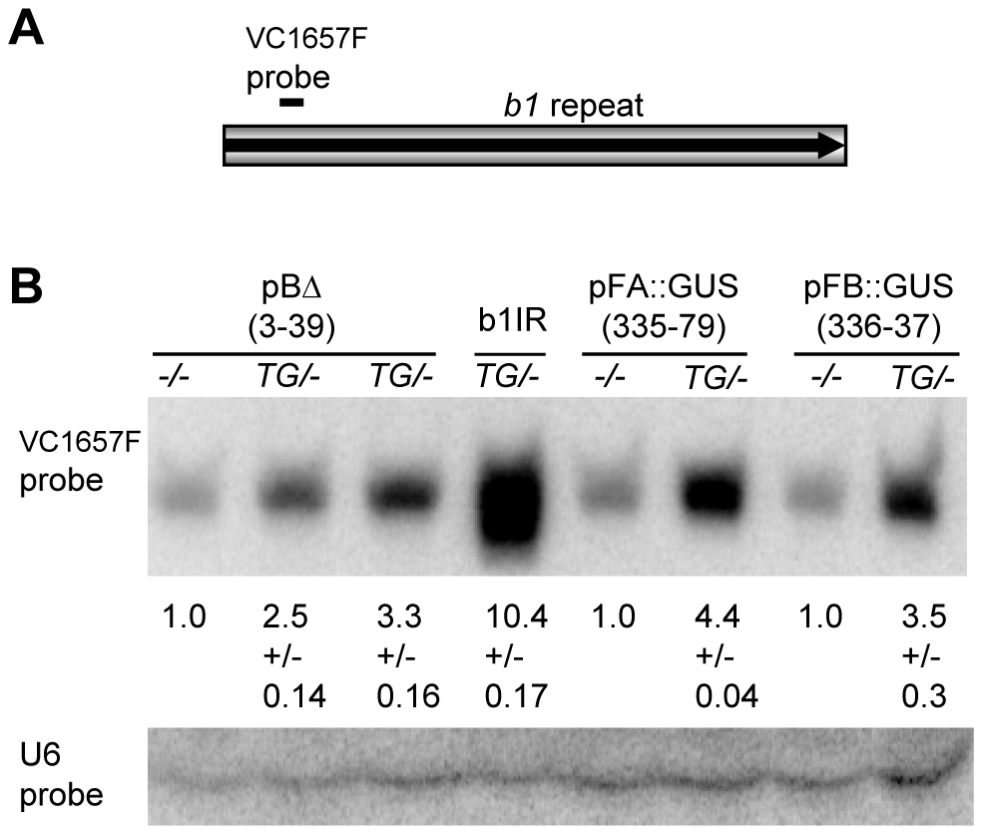
Northern blot analysis of repeat siRNAs in transgenic plants. **A**. The levels of the *b1* tandem repeat siRNAs were detected with the indicated oligo probe (VC1657), which hybridizes to the FA part of the repeat. **B**. Genotypes of the plants used for the analysis are shown above the blot with −/− denoting the absence of the transgene and *TG/-* indicating the presence of one copy of the transgene locus. b1IR stands for 35S::b1IR. The RNA levels were detected by hybridization with ^32^P labelled DNA/LNA oligonucleotide probes as described by [24]. The levels of the *b1* tandem repeat siRNAs detected with the VC1657 probe were normalized to U6 RNA levels, which served as a loading control [24]. The average abundances of *b1* repeat siRNAs are presented relative to the levels in their non-transgenic sibling plants, which were set to 1.0; +/− indicates the standard deviation. A 35S::b1IR transgene that produces high levels of siRNAs was used as a positive control for hybridization and is described in [24]. Transgenes pBΔ 3-39, pFA::GUS and pFB::GUS are described in Figure 1 and Figure S6, respectively.

Previous studies have shown that the tandem repeats in *B-I* and *B'* produce siRNAs [24]. Therefore various repeat transgenes were tested for the production of *b1* repeat siRNAs from their ectopic locations. As *b1* alleles that have a single copy of the repeat unit, and do not participate in paramutation, also produce *b1* repeat siRNAs [24], non-transgenic siblings with the same *b1* genotype as their transgenic counterparts were tested alongside (Figure 6). Transgenic pBΔ 3-39 plants with the full length repeats showed slightly increased levels (∼2–3 fold) of *b1* repeat siRNAs relative to their non-transgenic siblings, suggesting that either the transgenic locus was producing *b1* repeat siRNAs and/or it triggered an increase in the production of *b1* repeat siRNAs from the endogenous alleles. Similar increases in *b1* repeat siRNAs were seen with pFA::GUS and pFB::GUS transgenes (Figure 6 and Figure S6A). Notably, the *b1* oligoprobe used in this experiment hybridizes to the FA part of the repeats, indicating that, at least in the pFB::GUS event, the *b1* siRNAs detected are derived from the endogenous *b1* repeat sequences. In spite of similar siRNA levels, the pBΔ 3-39 and pFA::GUS transgenic events were paramutagenic, while the pFB::GUS transgenic event was not (Figure 2 and data not shown), suggesting that the increased production of siRNAs was not sufficient to establish paramutation. A similar lack of correlation with paramutagenic ability and production of siRNAs was previously reported for endogenous *b1* alleles [24].

### The maize *b1* tandem repeats do not display transcriptional regulatory activity in Arabidopsis

**Figure 7:**
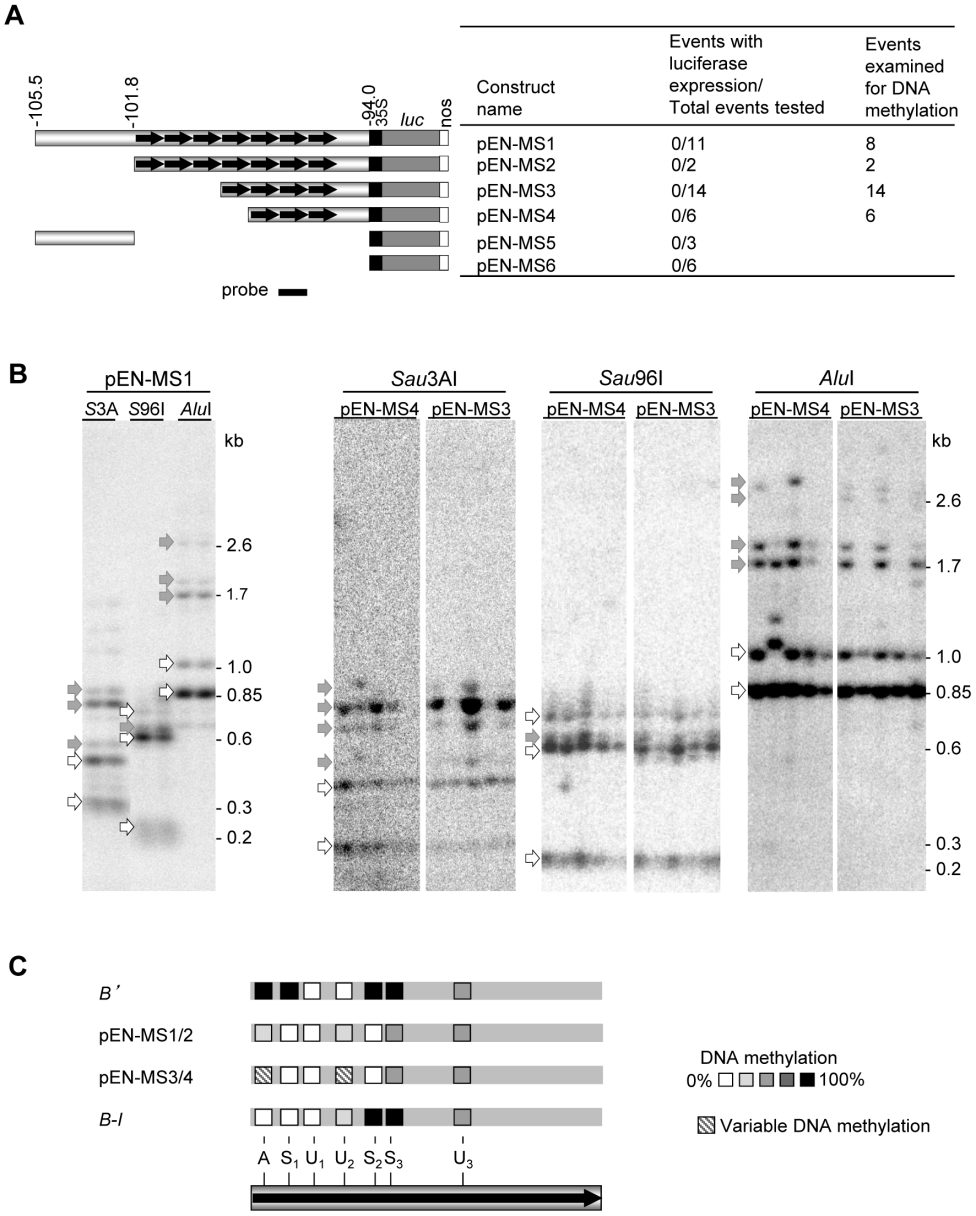
DNA methylation of maize *b1* repeats in *Arabidopsis*. **A**. Drawings of constructs used to transform *Arabidopsis*. The indicated *b1* fragments were fused to the minimal 35S promoter, the luciferase reporter gene and nopaline synthase (nos) polyadenylation signal. The numbers above the diagrams indicate the genomic location from where the *b1* sequences are derived relative to the *b1* transcription start site in kb. Every independent transgenic event with an intact insertion was tested for luciferase activity; the numbers are indicated. The number of these events also tested for DNA methylation is indicated as well. **B**. DNA methylation analyses of *b1* repeats in primary *Arabidopsis* transgenic plants. Genomic DNA was digested with *Eco*RI, which cuts on both sides of the repeats, and one of three methylation sensitive enzymes, *Sau*3AI, *Sau*96I or *Alu*I. Representative examples are shown for each enzyme combination. Additional examples are shown in Figure S7A. Open arrows indicate fragments derived from complete digestion (no DNA methylation), while gray arrows indicate fragments containing one or more undigested, cytosine methylated restriction sites. Fragment sizes are indicated on the right of the blots. **C**. Summary of the DNA methylation pattern of the *b1* tandem repeats in transgenic events, and *B'* and *B-I* for comparison [21]. The single repeat shown represents all repeats present in each transgenic event or allele; the *Sau*3A (A), *Sau*96A (S), and *Alu*I (A) restriction sites are indicated. Subscripts indicate individual recognition sites present more than once in each repeat. The methylation levels at each site are indicated by the gray-scale shown. The multiple independent pEN-MS1/2 transgenic events had similar levels of DNA methylation (solid shading). The pEN-MS3/4 transgenic events showed different DNA methylation levels at some sites in independent transgenic events (hatched shading). Figure S7 shows additional DNA blots and summarizes reporter gene assays for *b1* repeat transgenes in different *Arabidopsis* mutant backgrounds.

The observation that the *b1* tandem repeats are sufficient to recapitulate paramutation with a heterologous reporter gene in maize suggested that it might be possible to transfer the maize *b1* paramutation system to *Arabidopsis thaliana*. For Arabidopsis, a large set of well-characterized mutations affecting epigenetic regulation exist that could be tested for their involvement in paramutation. The first step was to generate transgenic loci in Arabidopsis that would be dependent on the *b1* tandem repeats for their expression. Constructs were generated with three to seven *b1* tandem repeats fused to the minimal −90 35S promoter and the luciferase reporter gene (Figure 7A). As a control, sequences upstream of the repeats (Figure 7A) or *b1* proximal promoter sequences (not shown) were used. Extensive analysis of the transgenic Arabidopsis plants containing intact transgenes revealed that all transgenic events carrying the *b1* repeats exhibited a low level of luciferase activity similar to that displayed by control events with no *b1* sequences (Figure 7A and Table S3). One possibility was that the transgenes integrated into a *B'*-like epigenetic state, which is associated with DNA methylation [19], [21]. Analyses of methylation using DNA blot analyses (Figure 7B and 7C, Figure S7A) revealed low levels of DNA methylation within the repeats and no detectable methylation in sequences upstream or downstream of the tandem repeats. All 7-repeat-containing transgenic events analyzed (pEN-MS1 and pEN-MS2) showed similar DNA methylation patterns compared to each other and also to that of the maize transgenes with seven repeats (Figure 4A). Such uniformity among transgenic events is unusual as methylation patterns between independent transgenic events are typically more variable [30]–[33]. The transgenic events carrying four and three *b1* repeats (pEN-MS3 and pEN-MS4) also displayed low methylation levels within the repeats, but there was more variation between the different independent transgenic events (Figure 7B and 7C, and data not shown), similar to that seen for the endogenous maize three-repeat allele [19]. Together, these results demonstrate that, in the primary transgenic plants, the transgenic repeat sequence acquired similar sparse DNA methylation in maize and *Arabidopsis*.

During the *Arabidopsis* transformation process *de novo* DNA methylation occurs [34], [35]. We hypothesized that preventing any DNA methylation from occurring may enable the detection of the transcriptional regulatory function of the *b1* repeats. To investigate this hypothesis, an Arabidopsis line in which the *de novo* DNA methyltransferases *drm1* and *drm2* (*DOMAIN REARRANGED DNA METHYLASE* 1 and 2; [34]) were mutated, was transformed with pb1::GFP constructs carrying *b1* repeat- or *b1* proximal promoter sequences fused to the minimal 35S promoter and GFP (Green Fluorescent Protein) coding region (Figure S7B). As a positive control, the 35S enhancer was fused to the GFP reporter gene (p35S::GFP). None of the pb1::GFP transgenic events showed GFP expression, while all of the p35S::GFP events did (Figure S7B and S7C, and Table S4). DNA blot analyses revealed that the *drm1 drm2* double mutant background did prevent DNA methylation within the *b1*-repeats (Figure S7D), indicating that the lack of GFP expression was not due to DNA methylation.

Two other possible explanations for a lack of GFP expression, RNA-directed transcriptional or post-transcriptional silencing, were tested using the appropriate *Arabidopsis* mutants. Constructs with either seven or three *b1* repeats (Figure S7B) were introduced into the *rdr2-1* [35] and *sgs2-1/rdr6* [36] mutants. *RDR2* mediates RNA-directed transcriptional gene silencing, and *RDR6* post-transcriptional gene silencing. None of the transgenic plants showed GFP expression (Figure S7B, Table S4), suggesting that neither RNA-directed transcriptional or post-transcriptional silencing is responsible for the lack of GFP expression. Taken together these data suggest that the maize *b1* repeats do not have transcriptional regulatory activity in *Arabidopsis*. As one needs transcription to study transcriptional silencing this approach is not viable to study paramutation in *Arabidopsis*.

## Discussion

Results of the transgenic analysis presented in this paper demonstrate that specific tandem repeats are sufficient to both send and respond to the paramutation signal and that the repeat sequences need not be in an allelic position to communicate. The *Mop1* gene, necessary for endogenous paramutation, is also required for transgene-induced paramutation, suggesting common mechanisms. The sequences required and sufficient for paramutation are localized in the first half of the *b1* repeat unit. The tandem repeats are furthermore sufficient to enhance the expression of a heterologous reporter gene in maize, but not *Arabidopsis*. While transgenes are capable of inducing paramutation, several key differences exist between endogenous- and transgene-induced paramutation. Endogenous *b1* paramutation is stable, fully penetrant and associated with dense DNA methylation within the *b1* repeats, while transgene-induced paramutation displays variation in stability, penetrance and DNA methylation levels within the transgenic and endogenous *b1* repeats.

Repeats have been implicated in multiple examples of paramutation [5], [19], [37], [38] and other silencing phenomena (e.g. [39]–[41]), but detailed mechanisms for why multiple copies are quantitatively required is not known in any system. Multiple models postulating which properties of the repeats are being counted have been discussed (reviewed in [42]). Models include a quantitative increase in a repeat product such as siRNAs [43], the quantitative binding of regulatory proteins to the repeats [44], the extent of DNA methylation within the repeats [21], or the creation and amplification of a unique junction fragment [21]. The transgenes were able to slightly elevate the production of siRNAs in immature ears but as we previously observed [24] there was no correlation between levels of siRNAs and the ability to participate in paramutation. These results do not exclude the possibility of a correlation between repeat siRNA levels and paramutation in other tissue types and/or developmental timepoints. Our results that tandem repeats of either the full repeat unit or the FA half are both strongly paramutagenic, yet they have distinct junctions, argues against a critical role for the junction regions. Furthermore, our observation that multiple repeats of FB have no paramutation activity strongly suggests tandem repeats of a specific sequence within FA are being counted during paramutation.

The FA and FB fragments differ in several properties that could be contributing to their ability to mediate paramutation. The FA half is much more GC rich relative to FB and as such, it contains most of the differentially DNA methylated region, including “the seed region” which becomes methylated very early in development in plants undergoing endogenous paramutation [21]. One possibility is that the AT richness of FB (68%) and the resulting lower capacity for cytosine methylation may prevent it from receiving and/or transmitting silencing signals. Intriguingly, the FA transgenes tended to be more strongly paramutagenic than those with the full repeat, suggesting that removal of the FB sequence increases the strength of paramutation. A full repeat is likely to have a lower overall density of DNA methylation than an FA repeat, which could be the signal being counted. It is also possible that FA, but not FB contains the regulatory sequences necessary to generate RNA silencing signals. The endogenous FB sequence is transcribed at a lower level and produces lower amounts of siRNAs relative to FA [24], [45]. Even though FB is neither required nor sufficient for paramutation in the transgenic assay, it may contribute to endogenous paramutation. Support for this hypothesis is that overexpression of a protein that binds to FB can induce a heritable and paramutagenic silenced state at the endogenous *B-I* allele [44]. Future experiments such as further dissecting the minimal sequences required for paramutation, mapping the key sequences mediating transcription of the *b1* repeats and characterization of additional DNA binding proteins, should help to distinguish between hypotheses.

Two broad classes of models have been proposed for the allelic interaction that mediates endogenous paramutation, diffusible *trans*-acting signals or pairing between the repeats - these models are not mutually exclusive. Our observation that many different transgenic loci, located at distinct genomic sites, efficiently induce paramutation is most consistent with a diffusible *trans*-acting signal mediating the initial communication establishing paramutation. Consistent with this hypothesis, mutations in multiple genes involved in the RNA-directed transcriptional silencing pathway prevent the establishment of paramutation (reviewed in [42]), suggesting RNA may be the signal. However, our transgene experiments do not eliminate repeat pairing, as there are examples of pairing between homologous sequences in non-allelic positions in other systems [46]–[48]. Future experiments employing cytological methods may be able to shed light on whether there is a role for DNA pairing in paramutation.

Fine structure recombination mapping and chromosome conformation capture studies demonstrated that the *b1* tandem repeats are also required for transcriptional activation of *b1* [19], [22], but those studies could not distinguish between a direct role, *i.e.* the repeats carry transcriptional regulatory sequences, versus an indirect role, i.e. they mediate the ability of regulatory sequences located elsewhere to activate *b1*. Our maize transgenic results demonstrate that the *b1* repeats do carry sequences that can mediate transcriptional activation of heterologous reporter genes, most consistent with a direct role of the repeats in transcriptional activation. Previous chromatin immunoprecipitation experiments demonstrated that upon transcriptional activation of *B-I*, the repeats are relatively depleted for nucleosomes and those that remain are enriched for H3ac histone marks [21]. These two properties, which strongly correlate with active transcriptional regulatory sequences [49], [50], are observed in both the FA and FB halves [21].

There is only one other paramutation system (*p1, pericarp color*) in which the sequence mediating paramutation has been defined [5], and that sequence also contains transcriptional regulatory activity [51], [52]. However, simply having a transcriptional regulatory element is not sufficient for paramutation as there are two transcriptional regulatory elements at *p1* and only one of them can induce paramutation [5]. In contrast to the observations in maize, the *b1* tandem repeats did not function as a transcriptional activator in *Arabidopsis*, suggesting that the transcription factors recognizing this sequence are not conserved between maize and *Arabidopsis*.

When *B-I* is paramutated by the repeat transgenes, the resulting transgene-induced *B'^#^* state, while heritable, often induced paramutation at a lower frequency and was less stable relative to the endogenous *B'* allele-induced *B'* state, in spite of the sequences being identical. The fact that after the transgenes are crossed to *B'*, they induced a much more stable *B'^#^* state, indicates that their non-allelic positions or the structure of the transgenic loci cannot be responsible for the original reduced penetrance and heritability. Furthermore, the observation that a generation together with *B'* increased the transgenes' paramutagenicity, relative to carrying the transgenes over neutral alleles, suggests some type of heritable epigenetic mark is accumulating. Precedence for a role for DNA methylation has been reported in *Arabidopsis* where the RNA-directed transcriptional silencing machinery requires the presence of pre-existing DNA methylation on the endogenous *FWA* locus for effective silencing of an incoming *FWA* transgene [40]. This may not be the case with paramutation in maize, as two transgenes with very low DNA methylation levels could induce paramutation of the endogenous allele. Our results do indicate that specific sequences within the FA region of the repeat are a critical component and given that most of the DNA methylation marks are within this region, it remains possible that DNA methylation marks contribute to the strength of paramutation. Further studies of multiple transgenic events will be required to test this hypothesis.

## Materials and Methods

### Maize seed stocks

The *b1* stocks were initially acquired from a variety of sources and have been maintained in the Chandler laboratory for a number of years. The *B'*, *B-I* and neutral *b1* alleles were obtained from E.H. Coe, Jr. (University of Missouri, Columbia) and *B-P* was obtained from M.G. Neuffer (University of Missouri). All maize plant stocks used in this study carry functional alleles for all biosynthetic genes and the other regulatory genes required for anthocyanin biosynthesis, unless otherwise indicated. All genetic tests were conducted in the irrigated field conditions in Tucson, Arizona.

### *Arabidopsis* seed stocks

The seed stocks used were wild type *Arabidopsis thaliana* (ecotype WS) and the previously described mutants *drm1 drm2* (ecotype Ws-2; [34]), *rdr2* (ecotype Col-0, SAIL_1277H08; [35]) and *rdr6* (*sgs2*, Col-0 [36]). All *Arabidopsis* plants were grown under standard greenhouse conditions.

### Maize plasmid and BAC clone construction

The pB clone (Figure 1A) contains 106.6 kb of sequences upstream of the *b1* transcription start site plus exon one, two and part of exon three (also named pBACB'1 in [20]; accession AY078063). The pBΔ clone was produced by digesting pB with the *Swa*I restriction enzyme, removing 91.6 kb of internal sequences and religation of the remaining sequences [20]. To produce the pFA and pFB transgenes, the two halves of the repeat were PCR amplified and inserted one by one in the *Bam*HI/*Bgl*II digested P1.0b::GUS plasmid [51]. The p35S::GUS construct (Figure 5A) was the same as −90 35S::GUS described in [53] and contained the minimal −90 bp Cauliflower Mosaic Virus 35S promoter (35S), the maize *adh1*gene intron1, the omega leader, the beta-glucuronidase (GUS) coding region, and the potato *PinII* terminator. To produce the pb1TR::GUS construct, the seven 853 bp repeat array was inserted in the p35S::GUS construct upstream of the 35S promoter. To produce the pFA::GUS and pFB::GUS constructs, the FA and FB tandem repeats were ligated upstream of the 35S promoter of the p35S::GUS construct, respectively. Primer information and detailed information on cloning and vectors used for plasmid construction is presented in the Methods S1.

### Maize transformation

Transgenic maize plants were generated at the Iowa State University Plant Transformation Facility using biolistic particle bombardment of *Hi-II* immature embryos, which carry a neutral *b1* allele (*b-N*) [54], [55]. The plasmid pBAR184 carrying the BAR gene, which confers resistance to the herbicide bialaphos, was co-bombarded with each construct [55]. Herbicide resistant calli were screened for DNA of interest using DNA blot analysis. Transformation events carrying transgene copies of the *b1* repeat DNA were regenerated from calli.

### *Arabidopsis* plasmid construction

The first set of plasmids used for *Arabidopsis* transformation carried the luciferase reporter gene (Figure 7A). These plasmids were made by inserting fragments of the maize *b1* gene in front of a −90 35S promoter fused to the omega leader, luciferase coding region and nopaline synthase (nos) polyadenylation signal. The second set of the plasmids contained a GFP reporter gene (Figure S7). These plasmids were produced either by replacing the luciferase reporter gene by a GFP reporter gene from the pFLUAR100 plasmid [56] or by transferring the *b1* sequences to an intermediate plasmid containing the 90 bp-35S promoter-GFP-nos gene cassette. A detailed description of the cloning steps and vectors used for plasmid construction is provided in the Methods S1.

### *Arabidopsis* transformation and expression analysis

*Arabidopsis* plants were transformed as described by [57] using 5% sucrose, 0.05% Silwet L-77, 0.5× Murashige & Skoog basal salts (micro and macro elements; Duchefa). The dipped plants were covered with Saran wrap, placed in the dark the first night and then grown in the greenhouse to maturity. To screen for transgenic plants, depending on the binary vector used, fluorescent seeds were either selected using the Leica MZ FLIII stereo fluorescence microscope with a dsRed filter or seedlings were sprayed with 0.5% BASTA (Glufosinate) twice, two and three weeks after sowing in soil, and surviving plants were transferred to individual pots. Transgenic plants were examined for reporter gene expression. Luciferase activity was evaluated using the Luciferase Assay System (Promega) and GFP activity was examined using the Leica MZ FLIII stereo fluorescence microscope with a GFP2 and GFP3 filter.

### DNA extraction and DNA blot analysis

Transgenic maize calli were ground in liquid nitrogen and incubated with extraction buffer (200 mM Tris-HCl pH 7.5; 250 mM NaCl; 25 mM EDTA pH 8.0; 0.5% SDS) for 10 minutes, followed by phenol∶chloroform (1∶1) and chloroform extraction. DNA was precipitated with 1/10 of the volume of 3 M NaOAc and an equal volume of isopropanol. Pelleted DNA pellet was washed with 70% ethanol and resuspended in TE (10 mM Tris-HCl pH 8.0; 1 mM EDTA). DNA extraction from maize leaves and *Arabidopsis* flower heads was performed according to [58], [59], respectively. For DNA blot analysis 4–5 µg of maize and 0.5–2.5 µg of *Arabidopsis* genomic DNA was digested with the appropriate restriction enzyme(s) following the manufacturer's specifications, size-fractionated by electrophoresis in 0.5× TBE 0.8–1.5% agarose gels, transferred to positively charged nylon membranes, fixed by UV fixation and hybridized with ^32^P labeled DNA probes as described [26]. All blots that contained samples digested with DNA methylation sensitive enzymes were probed with a fragment (Probe A [19]) that recognizes sequences that are not methylated in maize to confirm all restriction enzymes cut the DNA to completion [19] followed by hybridization to the *b1* repeat probe. Details describing probe fragments and restriction enzymes used for DNA blot analysis of maize and *Arabidopsis* transgenes are in Methods S1. Copy number of *b1* repeat units in maize transgenic plants was estimated using the software packages Quantity One (Biorad) for pB and pBΔ, and ImageJ [60] for pFA and pFB. Copy number was calculated and normalized to the intensity of a single copy band of one the endogenous *b1* allele present in each lane. Description of PCR-based genotyping of the endogenous *b1* alleles and the *mop1-1* mutation is presented in Methods S1.

### RNA extraction and Northern blot analyses

Small RNA fractions were extracted from young, immature (∼5 cm) maize ears as described by [24]. RNA was separated on denaturing polyacrylamide gels, hybridized with ^32^P end-labeled DNA/LNA *b1* repeat (VC1657F, [24]) and U6 (5′-CGTGTCATCCTTGCGCAGGGGCCATGCTAATCTTCTCTGTATCGT-3′) oligos. Results were analysed similarly to described previously [24].

### Analysis of GUS expression in maize transgenic plants

Tissues from transgenic plants (Figure 5 and Figure S6) were collected between ∼50–60 days after germination and incubated with 1 ml of 0.1% X-GLUC solution (5-bromo-3-chloro-2-indolyl-b-D-glucuronic acid, Sigma) in the dark at 37°C for 24 hours [52]. Chlorophyll pigment was removed by repeated incubations in 70% ethanol. Stained tissues were analyzed under a binocular microscope and categorized according to the staining levels shown in Figure 5B and Figure S6B.

## Supporting Information

Figure S1 Crossing scheme used to maintain transgenes in a neutral *b-N* background and to test transgene-induced silencing of *B-I*. Regenerated transgenic plants were crossed with non-transgenic *B-I/b-N* tester lines. Progeny carrying the *B-I* allele and transgene, which had been in the presence of a neutral *b1* allele for one generation, was assayed for transgene-induced silencing. Transgenic progeny plants carrying only neutral alleles (*b-N*) were outcrossed to plants carrying *b-N* for further maintenance in a neutral *b1* background and to *B-I* for testing transgene-induced silencing. Summaries of the *B-I* silencing tests are shown in Figure 2B and Table S2. Detailed data on transgene-induced silencing over multiple generations of propagation with neutral *b1* alleles is shown in Table S1.
(TIFF)

Figure S2 Crossing scheme to test for the heritability and paramutagenicity of the *B'^#^* state induced by pBΔ and pFA transgenes. In all testcrosses, genotyping was used to distinguish segregating *b1* alleles and to identify the presence/absence of a transgene. **A**. To assay heritability and paramutagenicity of the silenced *B'^#^* state after one generation of exposure to a transgene, transgenic plants displaying a *B'^#^* silencing phenotype were crossed with a plant heterozygous for the paramutable *B-I* and a neutral *b-N* allele. The phenotypic data for the informative progeny classes are presented in Table 1 ^b and 1d^. *B'∧* is used to indicate a *B-I* allele silenced by *B'^#^*. **B**. To assay heritability and paramutagenicity of *B'^#^* after two generations of exposure to a transgene, transgenic *B'^#^/b-N* plants from the first testcross were crossed with plants carrying either *b-N* (heritability test) or *B-I* (paramutagenicity test). Phenotypic data on informative progeny classes are presented in Table 1 ^c^ and 1^e^.
(TIFF)

Figure S3 Crossing scheme to test whether exposure to *B'* increases the silencing potential of weakly paramutagenic or non-paramutagenic pB, pBΔ and pFB transgenic events. The *b-N/b-N; TG/-* F_1_ (indicated in Figure 2A) of fourteen independent transgenic events that were initially not paramutagenic and four events that were weakly paramutagenic were crossed to *B'* plants. Transgenic progeny plants derived from these crosses were crossed with *B-I* to segregate the transgene from *B'* and to assay paramutagenicity of the transgenic events. The data are summarized in Table 2.
(TIFF)

Figure S4 Developmental profile of *b1* repeat DNA methylation pattern in a plant in which *B-I* is spontaneously changing to *B'*. **A**. Map of restriction sites and probe used for DNA blot analysis; *Eco*RI (E), *Alu*I (A), and *Sau*96I (S). Subscripts indicate specific recognition sites present more than once each repeat. **B**. Seeds were planted from a family showing a high frequency of spontaneous paramutation. Leaves were taken from plants at different stages of development. Representative examples of some developmental stages are shown in the diagram. **C**. DNA methylation was assayed in leaves collected at different stages of plant development (see panel B). Leaf DNA was digested with the methylation insensitive enzyme *Eco*RI and the cytosine methylation sensitive enzymes *Alu*I or *Sau*96I. DNA blots were probed with the *b1* repeat probe indicated in panel A. As a control, the *B-I* and *B'* DNA methylation profile is shown for each enzyme. Open arrows indicate completely digested DNA. Gray arrows indicate fragments in which the S2 and S3 sites are methylated, while black arrows indicate fragments in which all sites are methylated. The fragments in between the open and black arrows are the result of DNA methylation at one or more of the assayed sites within the *b1* repeats.
(TIFF)

Figure S5 Crossing scheme for testing whether the *mop1-1* mutation can prevent the pBΔ transgenes from paramutating *B-I*. The plus sign denotes the wild-type *Mop1* allele. Transgenic *b-N mop1-1/B-P+*plants were crossed to homozygous *B-I mop1-1* plants and the colorless seeds planted. *B-P* is a neutral *b1* allele and provides purple seed color that was used to identify seeds carrying this allele. Because *mop1-1* was linked to *b-N* in the transgenic plants, the majority of the progeny plants were homozygous for the *mop1-1* mutation (parental, non-recombinant classes, 77%) and a minority was heterozygous for the mutation (recombinant classes, 23%). Molecular genotyping was used to distinguish between the segregating progeny classes. In *mop1-1* homozygotes, *B'* expression is up-regulated resulting in dark plant phenotypes [62]. To determine whether paramutation of *B-I* occurred in these plants, testcrosses were done with a neutral *b1* allele (*b-N*) that specifies no plant pigment. The resulting progeny were genotyped for transgene presence and assayed for plant pigment. The data on the informative progeny class is shown in Table 3.
(TIFF)

Figure S6 GUS staining in leaves and sheath of pFA::GUS and pFB::GUS transgenic events. **A**. Drawing of the pFA::GUS and pFB::GUS constructs with the sequence components indicated on the top. All components, except the *b1* sequences, were the same as those described for the constructs shown in Figure 5 (see also Materials and Methods). **B**. The scoring scale used to evaluate GUS expression levels in leaves and sheath tissue of transgenic plants. **C**. Chart showing percentages of transgenic plants with the GUS staining levels indicated in panel B. Leaf and sheath tissues are denoted as L and S, respectively. The number of plants assayed is shown on top of each pair of columns.
(TIFF)

Figure S7 *Arabidopsis b1-*repeat::*luciferase* transgenes carrying seven tandem repeats display low levels of DNA methylation. **A**. DNA blot analyses for the pEN-MS1 and pEN-MS2 transgenes. Genomic DNA was cut with the methylation insensitive enzyme *Eco*RI (E) and one of three methylation sensitive enzymes, *Sau*3AI (U), *Sau*96I (S) and *Alu*I (A). Open arrows indicate fragments derived from complete digestion of genomic DNA, while grey arrows indicate fragments consistent with the presence of cytosine DNA methylation in one or more restriction sites within the *b1* repeats. Each lane contains DNA of an independent transgenic line. **B**. The indicated *b1* fragments were fused to the minimal 35S promoter and the GFP reporter gene. The numbers above the diagrams indicate the location (in kilobases) from which the sequences are derived relative to the *b1* transcription start site. The minimal −90 bp 35S promoter region contained no enhancer sequences [63]. The pRB7 plasmid was used to verify the functionality of the GFP reporter gene and carried 747 bp of the 35S enhancer sequence. The number of independent transgenic events that were tested for GFP expression is indicated. **C**. Representative photos of pRB7 and pRB1 transgenic events. The greenish color of the pRB7 inflorescence is characteristic for GFP expression. There was no detectable green fluorescence in the pRB1 inflorescence. The observed reddish color is due to chlorophyll autofluorescence. **D**. DNA methylation analyses of pRB1 transgenic *drm1 drm2* plants. Genomic DNA was cut with *Eco*RI and *Alu*I and the resulting blot hybridized with the *b1* repeat and a GFP probe. A few representative samples are shown. The complete digestion observed with the *b1* repeat probe indicates that the *drm1 drm2* double mutation prevented DNA methylation of the *Alu*I sites. The hybridization with a GFP probe demonstrates that the DNA was digested to completion.
(TIFF)

Methods S1 The Methods S1 describe the construction of plasmids used for maize and *Arabidopsis* transformation, restriction enzymes and probes used for Southern blot analyses of *Arabidopsis* and maize plants, and genotyping assays used in experiments that tested the requirement of MOP1 for transgene-induced paramutation.
(DOCX)

Table S1 Frequency of pB and pBΔ-induced *B-I* silencing.
(DOCX)

Table S2 Frequency of transgene-induced *B-I* silencing after propagation with a neutral *b1* allele.
(DOCX)

Table S3 DNA blot analysis of the *Arabidopsis* transgenes.
(DOCX)

Table S4 Analysis of effect of *b1* repeats on expression of GFP reporter gene in *Arabidopsis* mutants.
(DOCX)
