# Supplementary figures and images for: Specific Tandem Repeats Are Sufficient for Paramutation-Induced Trans-Generational Silencing
Source: PLoS Genet. 2013 Oct 17;9(10):e1003773. doi: 10.1371/journal.pgen.1003773 (PMC3798267; doi:10.1371/journal.pgen.1003773)

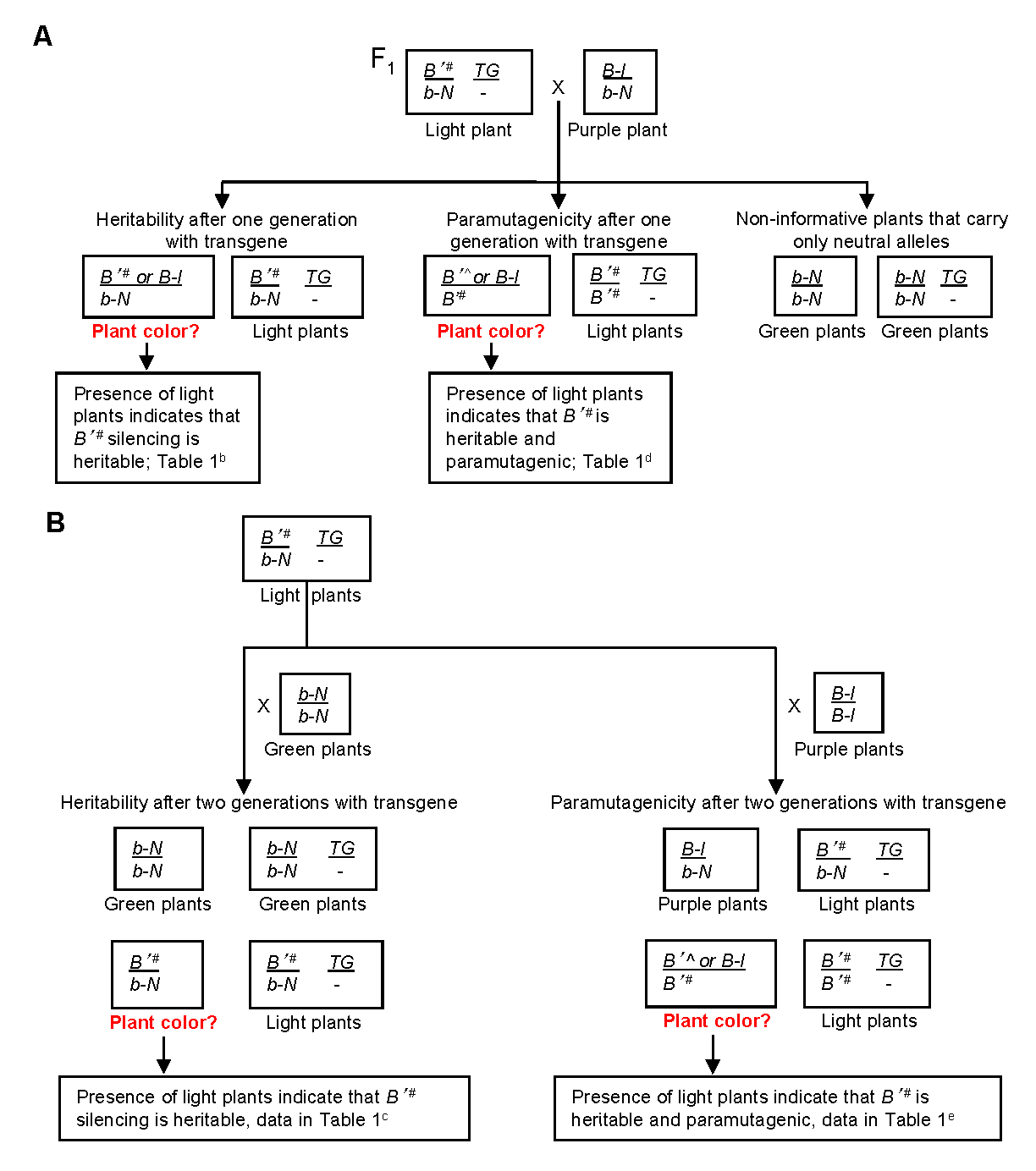

Supplement: Figure S2 — Crossing scheme to test for the heritability and paramutagenicity of the B'# state induced by pBΔ and pFA transgenes. In all testcrosses, genotyping was used to distinguish segregating b1 alleles and to identify the presence/absence of a transgene. A. To assay heritability and paramutagenicity of the silenced B'# state after one generation of exposure to a transgene, transgenic plants displaying a B'# silencing phenotype were crossed with a plant heterozygous for the paramutable B-I and a neutral b-N allele. The phenotypic data for the informative progeny classes are presented in Table 1 b and 1d. B'∧ is used to indicate a B-I allele silenced by B'#. B. To assay heritability and paramutagenicity of B'# after two generations of exposure to a transgene, transgenic B'#/b-N plants from the first testcross were crossed with plants carrying either b-N (heritability test) or B-I (paramutagenicity test). Phenotypic data on informative progeny classes are presented in Table 1 c and 1e. (TIFF) [file pgen.1003773.s002.tiff]

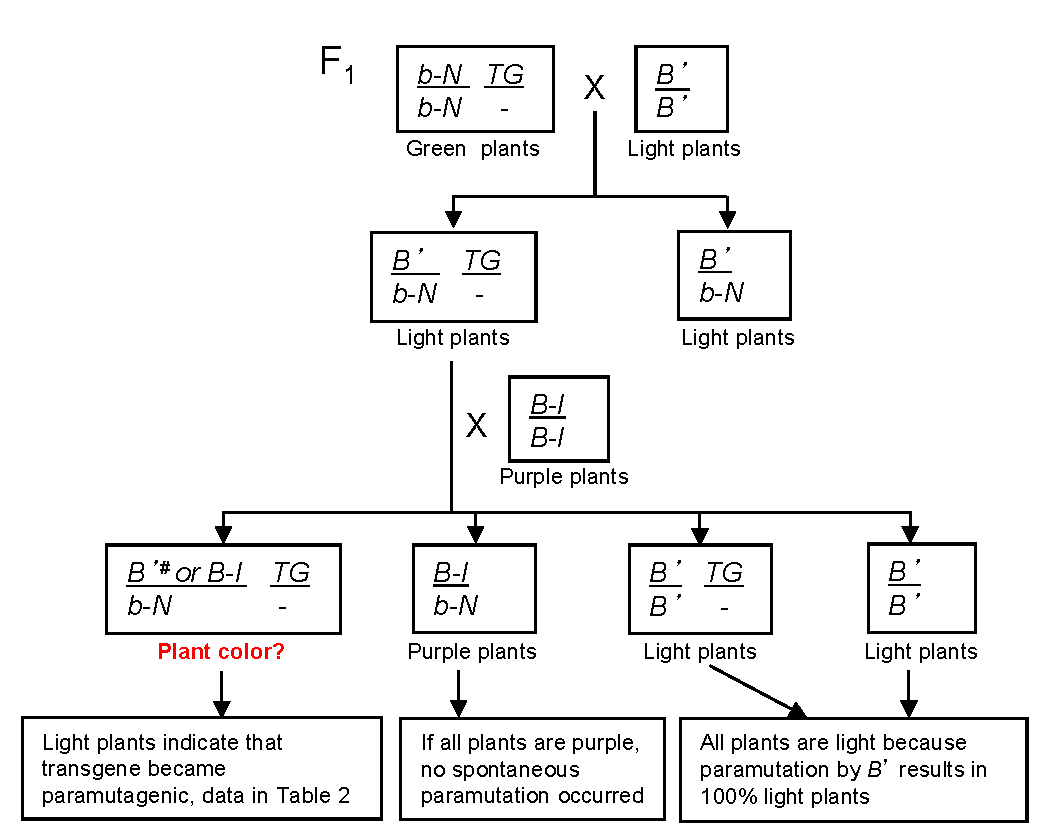

Supplement: Figure S3 — Crossing scheme to test whether exposure to B' increases the silencing potential of weakly paramutagenic or non-paramutagenic pB, pBΔ and pFB transgenic events. The b-N/b-N; TG/- F1 (indicated in Figure 2A) of fourteen independent transgenic events that were initially not paramutagenic and four events that were weakly paramutagenic were crossed to B' plants. Transgenic progeny plants derived from these crosses were crossed with B-I to segregate the transgene from B' and to assay paramutagenicity of the transgenic events. The data are summarized in Table 2. (TIFF) [file pgen.1003773.s003.tiff]

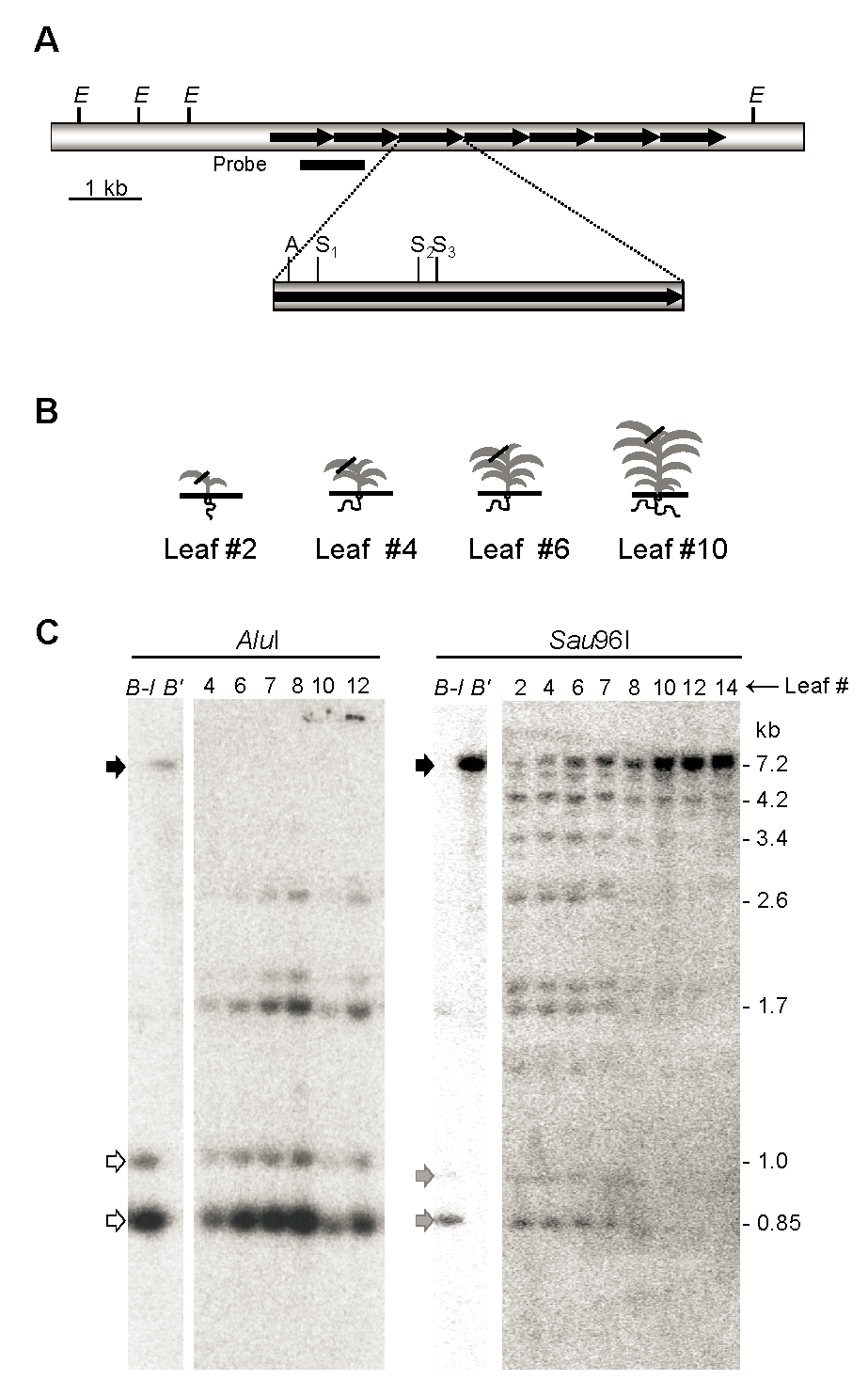

Supplement: Figure S4 — Developmental profile of b1 repeat DNA methylation pattern in a plant in which B-I is spontaneously changing to B'. A. Map of restriction sites and probe used for DNA blot analysis; EcoRI (E), AluI (A), and Sau96I (S). Subscripts indicate specific recognition sites present more than once each repeat. B. Seeds were planted from a family showing a high frequency of spontaneous paramutation. Leaves were taken from plants at different stages of development. Representative examples of some developmental stages are shown in the diagram. C. DNA methylation was assayed in leaves collected at different stages of plant development (see panel B). Leaf DNA was digested with the methylation insensitive enzyme EcoRI and the cytosine methylation sensitive enzymes AluI or Sau96I. DNA blots were probed with the b1 repeat probe indicated in panel A. As a control, the B-I and B' DNA methylation profile is shown for each enzyme. Open arrows indicate completely digested DNA. Gray arrows indicate fragments in which the S2 and S3 sites are methylated, while black arrows indicate fragments in which all sites are methylated. The fragments in between the open and black arrows are the result of DNA methylation at one or more of the assayed sites within the b1 repeats. (TIFF) [file pgen.1003773.s004.tiff]

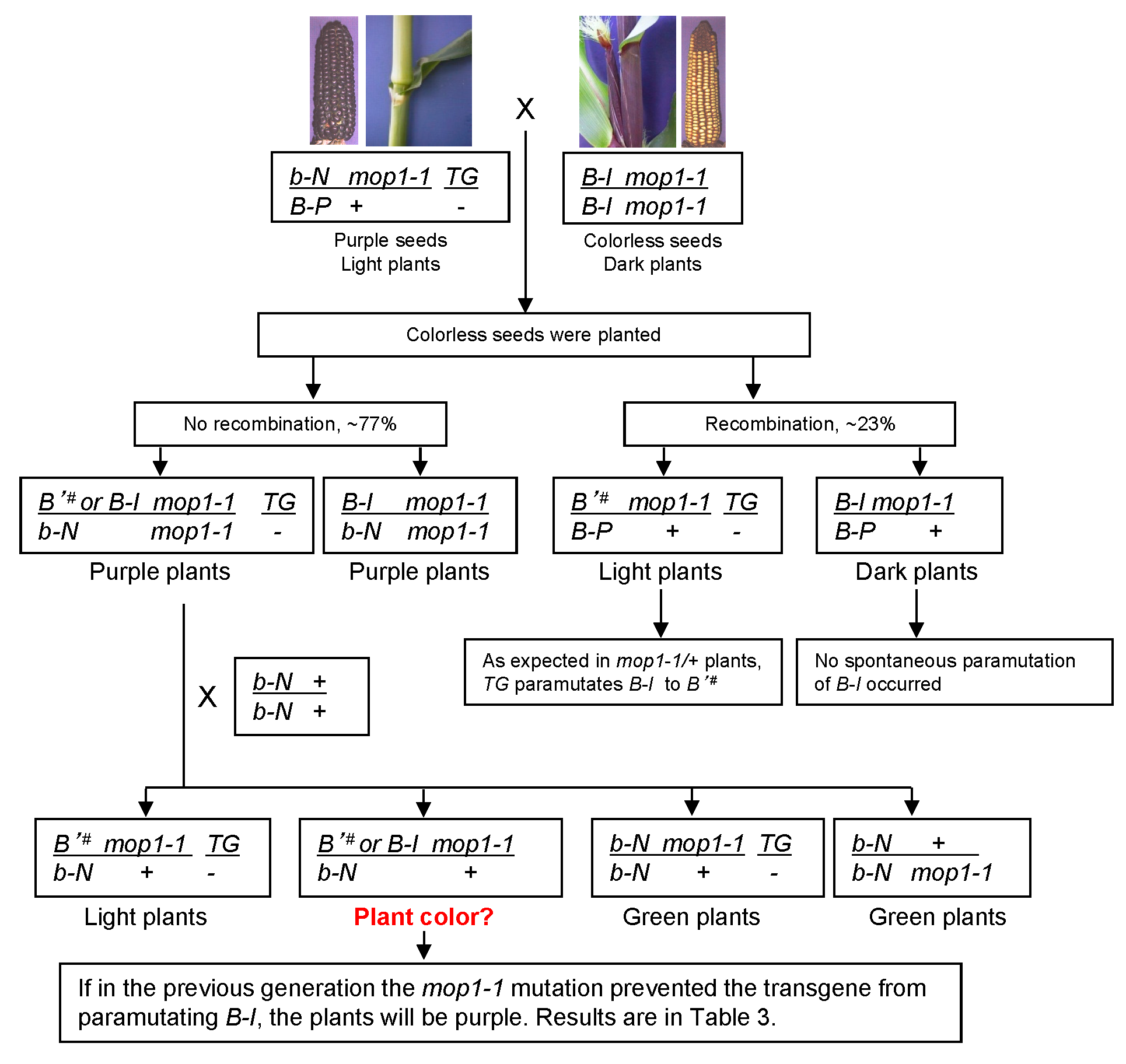

Supplement: Figure S5 — Crossing scheme for testing whether the mop1-1 mutation can prevent the pBΔ transgenes from paramutating B-I. The plus sign denotes the wild-type Mop1 allele. Transgenic b-N mop1-1/B-P+plants were crossed to homozygous B-I mop1-1 plants and the colorless seeds planted. B-P is a neutral b1 allele and provides purple seed color that was used to identify seeds carrying this allele. Because mop1-1 was linked to b-N in the transgenic plants, the majority of the progeny plants were homozygous for the mop1-1 mutation (parental, non-recombinant classes, 77%) and a minority was heterozygous for the mutation (recombinant classes, 23%). Molecular genotyping was used to distinguish between the segregating progeny classes. In mop1-1 homozygotes, B' expression is up-regulated resulting in dark plant phenotypes [62]. To determine whether paramutation of B-I occurred in these plants, testcrosses were done with a neutral b1 allele (b-N) that specifies no plant pigment. The resulting progeny were genotyped for transgene presence and assayed for plant pigment. The data on the informative progeny class is shown in Table 3. (TIFF) [file pgen.1003773.s005.tiff]

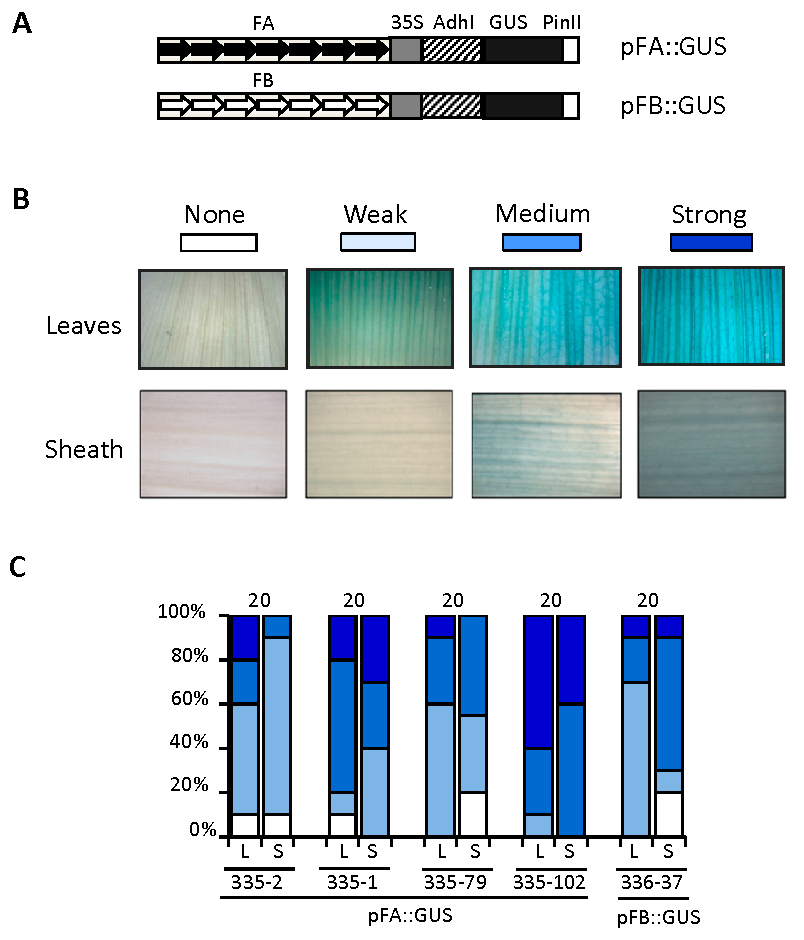

Supplement: Figure S6 — GUS staining in leaves and sheath of pFA::GUS and pFB::GUS transgenic events. A. Drawing of the pFA::GUS and pFB::GUS constructs with the sequence components indicated on the top. All components, except the b1 sequences, were the same as those described for the constructs shown in Figure 5 (see also Materials and Methods). B. The scoring scale used to evaluate GUS expression levels in leaves and sheath tissue of transgenic plants. C. Chart showing percentages of transgenic plants with the GUS staining levels indicated in panel B. Leaf and sheath tissues are denoted as L and S, respectively. The number of plants assayed is shown on top of each pair of columns. (TIFF) [file pgen.1003773.s006.tiff]

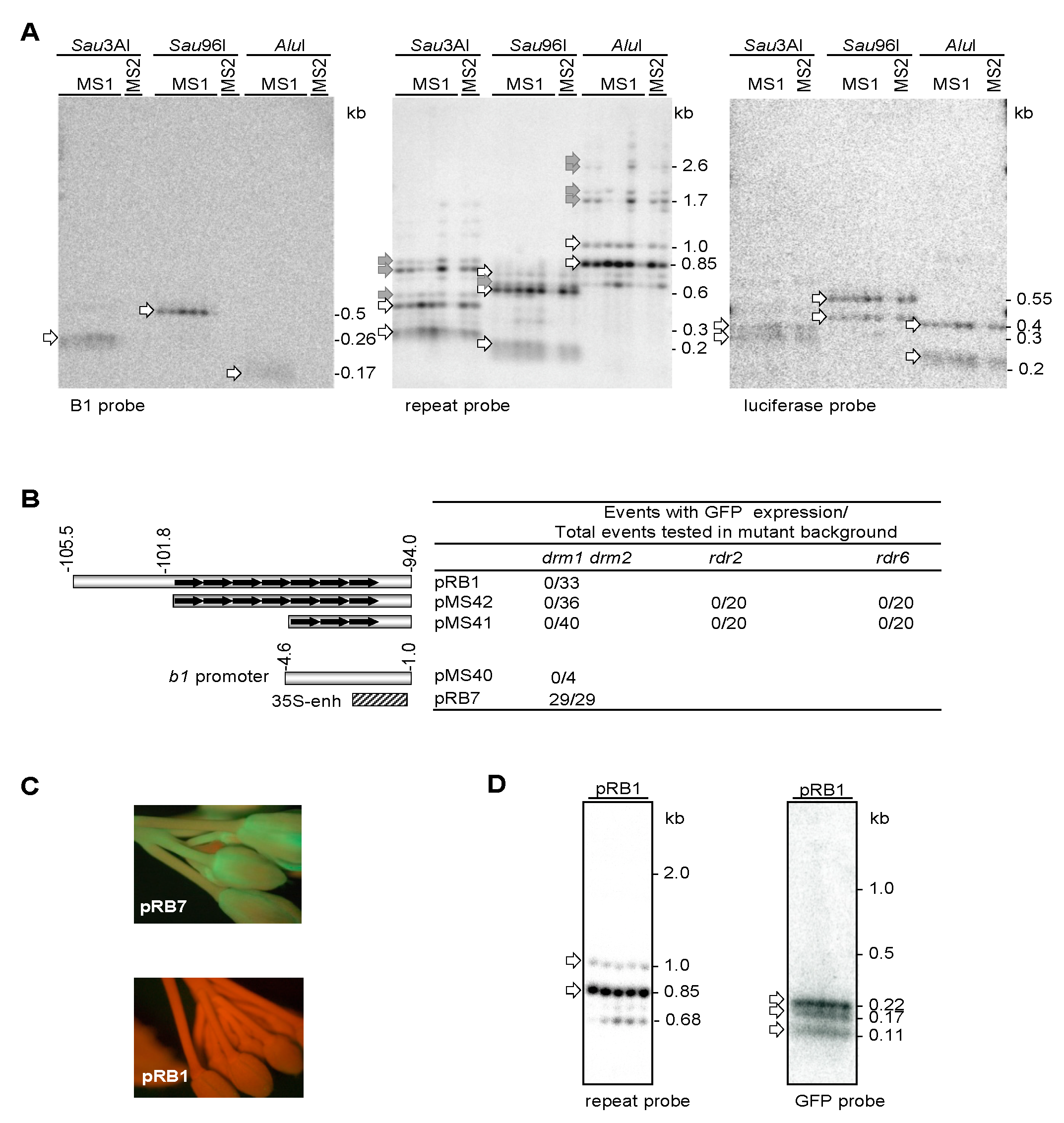

Supplement: Figure S7 — Arabidopsis b1-repeat::luciferase transgenes carrying seven tandem repeats display low levels of DNA methylation. A. DNA blot analyses for the pEN-MS1 and pEN-MS2 transgenes. Genomic DNA was cut with the methylation insensitive enzyme EcoRI (E) and one of three methylation sensitive enzymes, Sau3AI (U), Sau96I (S) and AluI (A). Open arrows indicate fragments derived from complete digestion of genomic DNA, while grey arrows indicate fragments consistent with the presence of cytosine DNA methylation in one or more restriction sites within the b1 repeats. Each lane contains DNA of an independent transgenic line. B. The indicated b1 fragments were fused to the minimal 35S promoter and the GFP reporter gene. The numbers above the diagrams indicate the location (in kilobases) from which the sequences are derived relative to the b1 transcription start site. The minimal −90 bp 35S promoter region contained no enhancer sequences [63]. The pRB7 plasmid was used to verify the functionality of the GFP reporter gene and carried 747 bp of the 35S enhancer sequence. The number of independent transgenic events that were tested for GFP expression is indicated. C. Representative photos of pRB7 and pRB1 transgenic events. The greenish color of the pRB7 inflorescence is characteristic for GFP expression. There was no detectable green fluorescence in the pRB1 inflorescence. The observed reddish color is due to chlorophyll autofluorescence. D. DNA methylation analyses of pRB1 transgenic drm1 drm2 plants. Genomic DNA was cut with EcoRI and AluI and the resulting blot hybridized with the b1 repeat and a GFP probe. A few representative samples are shown. The complete digestion observed with the b1 repeat probe indicates that the drm1 drm2 double mutation prevented DNA methylation of the AluI sites. The hybridization with a GFP probe demonstrates that the DNA was digested to completion. (TIFF) [file pgen.1003773.s007.tiff]
