# Supplementary material for: Specific Tandem Repeats Are Sufficient for Paramutation-Induced Trans-Generational Silencing
Source: PLoS Genet. 2013 Oct 17;9(10):e1003773. doi: 10.1371/journal.pgen.1003773 (PMC3798267; doi:10.1371/journal.pgen.1003773)
Supplement: Methods S1 — The Methods S1 describe the construction of plasmids used for maize and Arabidopsis transformation, restriction enzymes and probes used for Southern blot analyses of Arabidopsis and maize plants, and genotyping assays used in experiments that tested the requirement of MOP1 for transgene-induced paramutation. (DOCX) [file pgen.1003773.s008.docx]

**METHODS S1**

**Maize plasmid and BAC clone construction:** To produce the pFA and pFB transgenes, the two halves of the repeat, 413 bp and 467 bp respectively, were PCR amplified with primers that carried *BamH*I or *Bgl*II sites at their 5’-end. The resulting PCR fragment was digested with *BamH*I and *Bgl*II and cloned into *Bam*HI/*Bgl*II digested P1.0b::GUS plasmid [[1](#_ENREF_1)]. After the first repeat was cloned, additional repeats were inserted by ligating the *Bam*HI/*Bgl*II digested PCR fragment into a *Bgl*II digested vector from a previous step, until a seven tandemly repeated array was produced (Figure 1B). The forward and reverse primers used to amplify FA were: VC381 (5’- CGGGATCCCGCCATGGGTTTGCTGCATCCTTG -3’) and VC797 (5’- GAAGATCTTCGTGAAGGTAGTCTAAATATATAGG -3’). The primers to amplify FB were VC798 (5’- CGGGATCCCGCCTATATATTTAGACTACCTTCAC -3’) and VC382 (5’- GAAGATCTTCCCAAGTATTCGGTATAAAAGTTG -3’). To produce the pb1TR::GUS construct, the p35S::GUS construct [[2](#_ENREF_2)] was cut with *Xho*I, filled in with Klenow and re-ligated, producing the intermediate clone, pLP1046exXho. This clone was cut with *Asc*I and *Sal*I and an *Asc*I-*Sal*I-*Not*I-*Xho*I-*Bam*HI linker was inserted, producing pLP1046-oligo. The latter clone was cut immediately upstream of the -90-35S promoter with *Xho*I, filled in with Klenow, and then ligated to a 6.9 kb fragment derived from the pB∆ construct. This 6.9 kb fragment, containing the seven tandem repeats and 44 bp of upstream, and 863 bp of downstream flanking sequences, was prepared by cutting pB∆ with *Bam*HI, filling in with Klenow, and then digesting with *Bcl*I. To produce the pFA::GUS and pFB::GUS constructs, the FA and FB tandem repeats were released from pFA and pFB plasmids using *Bam*HI and *Bgl*II and ligated upstream of the -90 minimal 35S promoter, in the *Nsi*I-*Bam*HI-*Acc*I-*Bgl*II-*Asc*I-*Nsi*I linker inserted in the *Pst*I site of pLP1046. Detailed description of the vector backbone used in the pFA, pFB, pFA::GUS, pFB::GUS, p35S::GUS, and pb1TR::GUS plasmids is in [1].

**Arabidopsis plasmid construction:** To produce Arabidopsis transgenic plants carrying the *b1* tandem repeats a binary vector able to carry and mediate the transfer of large pieces of DNA, including repetitive sequences, was needed. The binary BAC vectors developed by Hamilton *et al.* [[3](#_ENREF_3),[4](#_ENREF_4)] were well suited for this purpose, but are hard to engineer. To facilitate the cloning of DNA of interest into a binary BAC vector, the Gateway ccdB cassette A (Invitrogen) was cloned into the *Not*I site of pCH20 [[3](#_ENREF_3)], resulting in a Gateway destination vector. To enable convenient selection of *Arabidopsis* transformants, a cruciferin promoter-driven *DsRed* gene (from pAR121, received from A.Stuitje) and a mas2’ promoter driven *Bar* gene (from pGSA1252; http://www.chromdb.org/rnai/pGSA1252.html) were inserted in the *Pac*I site of pCH20, resulting in BIBAC-RFP-GW and BIBAC-BAR-GW, respectively.

To enable recombination with the kanamycin resistant BIBAC-RFP-GW and BIBAC-BAR-GW destination vectors an entry vector carrying a resistance gene other than kanamycin had to be developed. To produce such a vector, the kanamycin resistance gene in a commercial entry vector (pENTR4, Invitrogen) was replaced with the gentamycin resistance gene from pDONR207 (Invitrogen). This required a BP recombination reaction, which is only possible between an expression vector and pDONR207. Therefore, pENTR4 was modified into an expression vector, pEXP4-Amp, in two steps. First, the ccdB cassette was removed from pENTR4, producing pENTR4ex-ccdB, which was then recombined with the destination vector pDEST14 (Invitrogen) in an LR reaction, creating pEXP4-Amp. This vector was capable of a BP reaction with pDONR207, creating the gentamycin resistant pENTR-gm entry vector.

The transgenic expression cassettes shown in Figure 7 and Figure S7 were cloned into the pENTR-gm. The pEN-MS, pMS and pRB expression cassettes contained upstream *b1* sequences, *b1* proximal promoter sequences, a 759 bp CaMV enhancer sequence (Figure S7), or no sequence in front of a 90-bp minimal CaMV-35S promoter fused to the omega leader, the coding region of a reporter gene and the nopaline (nos) polyadenylation signal. The reporter genes used were luciferase from pBS-LUC and enhanced green fluorescent protein from pFLUAR100 [[5](#_ENREF_5)]. The expression cassettes were inserted between the Gateway ATTL1 and ATTL2 recombination sites in the pENTR-gm vector.

The produced entry vectors were recombined in an LR reaction with the BIBAC-RFP-GW or BIBAC-BAR-GW destination vectors, and the resulting plasmids were transformed to *Agrobacterium tumefaciens* C58C1 carrying the pCH32 helper plasmid [[3](#_ENREF_3)]. Maps and details of how the constructs were generated are available upon request.

**Probes used for DNA blot analysis:** Fragments of maize *b1* upstream sequences, the luciferase, GFP and GUS gene fragments were labeled with ^32^P and used for hybridization with DNA blots. The *b1* 853 bp repeat probe was amplified with the M13 forward and reverse primer using pMS2, containing one repeat fragment, as a template. The FA and FB probes were the same FA and FB sub-fragments used for cloning (see above). The Probe A and Probe B1 described in [[6](#_ENREF_6)]. The luciferase probe is a 1209 bp *Eco*RI fragment containing most of the luciferase coding region except the first 589 bp. For the GFP probe, the entire GFP coding region, isolated from pFLUAR100 by cutting with *Nco*I and *Bam*HI, was used. The GUS probe is a 787 bp PCR fragment amplified from the 5’ end of the GUS coding region using the primers M83 (5’-TCCTGTAGAAACCCCAACCCG-3’) and M84 (5’-GTAGATATCACACTCTGTCTGG-3’) and pBI221 as a template [[7](#_ENREF_7)].

**DNA blot analysis of maize transgenes:** To select transgenic pB and pB∆ maize calli containing insertions of intact seven tandem repeats, DNA was digested with *EcoR*I and screened for the presence of the corresponding ~7 kb fragment. To verify the presence, copy number and segregation pattern of the transgenes, DNA blot analysis was repeated on DNA isolated from plants. Only integration events with a hybridization pattern identical to the transgenic calli and segregating as single transgenic loci were used in genetic experiments. The 853 bp repeat copy number was determined using the Quantity One software package (BioRad). More specifically, for each transgenic plant the sum intensity of all transgenic bands was divided by the intensity of a fragment containing the single repeat unit of a neutral *b1* allele present in the same plant (Figure 3). The p35S::GUS, pb1TR::GUS, and pFB::GUS, and pFA::GUS calli were screened by DNA blot analysis for the presence of the expression cassettes. For this, genomic DNA was digested with restriction enzymes flanking the expression cassettes. To determine the intactness and segregation of the p35S::GUS, pb1TR::GUS transgenes, DNA isolated from the progeny was digested with *Eco*RI and the blots hybridized with the GUS and repeat probe. The GUS probe recognized a discrete 3531 bp fragment if the GUS coding region was intact. The repeat probe provided an estimation of the number of transgenic insertions. The intactness of the transgenic inserts was further characterized by DNA blot analysis using *Nco*I single, and *Bam*HI, *Eco*RI double digests.

**Genotyping the endogenous *b1* alleles and the *mop1-1* mutation:** Endogenous *b1* alleles were genotyped by either DNA blot or PCR analyses. For DNA blot analysis genomic DNA was digested with *Nco*I and hybridized with the Probe A [[6](#_ENREF_6)] located upstream of the *b1* tandem repeats, producing a ~6 kb band for *b-N*, 5.6 kb band for *B-P* and a 6.8 kb band for *B-I* and *B’*. For PCR analysis genomic DNA was amplified with VC818 (5’-CAATAGGCACCCTCCACTTCGTGTC-3’) and VC 911 (5’-GATCGACGGTATGGGCCAGGGGAG-3’) to produce a ~200 bp band for *b-N* allele from *HiII*, a 204 bp band for *B-P* and a 362 bp band for *B-I* and *B’*. The presence of the *mop1-1* mutation was determined using a tightly linked phi083 SSR (5’-ATTCATCGACGCGTCACAGTCTACT-3’ and 5’-CAAACATCAGCCAGAGACAAGGAC- 3’).

**REFERENCES**

1. Sidorenko L, Li X, Tagliani L, Bowen B, Peterson T (1999) Characterization of the regulatory elements of the maize *P-rr* gene by transient expression assays. Plant Mol Biol 39: 11-19.

2. Sidorenko LV, Chandler VL (2008) RNA Dependent RNA Polymerase is Required for Enhancer Mediated Transcriptional Silencing Associated with Paramutation at the Maize *p1* Gene. Genetics 180: 1983-1993.

3. Hamilton CM (1997) A binary-BAC system for plant transformation with high-molecular-weight DNA. Gene 200: 107-116.

4. Hamilton CM, Frary A, Lewis C, Tanksley SD (1996) Stable transfer of intact high molecular weight DNA into plant chromosomes. Proc Natl Acad Sci U S A 93: 9975-9979.

5. Stuitje AR, Verbree EC, van der Linden KH, Mietkiewska EM, Nap JP, et al. (2003) Seed-expressed fluorescent proteins as versatile tools for easy (co)transformation and high-throughput functional genomics in *Arabidopsis*. Plant Biotechnol J 1: 301-309.

6. Stam M, Belele C, Dorweiler JE, Chandler VL (2002) Differential chromatin structure within a tandem array 100 kb upstream of the maize *b1* locus is associated with paramutation. Genes Dev 16: 1906-1918.

7. Jefferson RA, Kavanagh TA, Bevan MW (1987) GUS fusions: beta-glucuronidase as a sensitive and versatile gene fusion marker in higher plants. Embo J 6: 3901-3907.
