# Supplementary material for: Specific Tandem Repeats Are Sufficient for Paramutation-Induced Trans-Generational Silencing
Source: PLoS Genet. 2013 Oct 17;9(10):e1003773. doi: 10.1371/journal.pgen.1003773 (PMC3798267; doi:10.1371/journal.pgen.1003773)
Supplement: Table S1 — Frequency of pB and pBΔ-induced B-I silencing. (DOCX) [file pgen.1003773.s009.docx]

**Table S1. Frequency of pB and pBΔ-Induced *B-I* Silencing^a^.**

|  |  | *TG/-* plants assayed (Frequency of light *B’^#^/ b-N; TG/-* plants) | | | | | |  |
| --- | --- | --- | --- | --- | --- | --- | --- | --- |
|  |  | Number of generations *TG/*- was maintained with a neutral *b-N* allele  before being tested for paramutation efficiency | | | | | |  |
| Construct | Transgenic  event | 1 | 2 | 3 | 4 | 5 | 6 | Total plants |

| pB | 4-07 | 6 (17%) | Nt | Nt | Nt | Nt | Nt | 6 |
| --- | --- | --- | --- | --- | --- | --- | --- | --- |
|  | 4-27 | 5 (40%) | Nt | 31 (32%) | 42 (0%) | Nt | 65 (89%) | 143 |
|  | 4-36 | 5 (20%) | Nt | Nt | Nt | Nt | Nt | 5 |
|  | 4-43 | 7 (14%) | Nt | 15 (80%) | 57 (7%) | 10 (30%) | Nt | 89 |
|  | 4-03 | 4 (0%) | 62 (0%) | 17 (0%) | Nt | Nt | Nt | 83 |
|  | 4-06 | 4 (0%) | 27 (0%) | 50 (0%) | 99 (0%) | Nt | Nt | 180 |
|  | 4-10 | 22 (0%) | 30 (0%) | Nt | Nt | Nt | Nt | 52 |
|  | 4-12 | 4 (0%) | Nt | 90 (0%) | 53 (0%) | Nt | Nt | 147 |
|  | 4-14 | 5 (0%) | Nt | 132 (0%) | 20 (0%) | Nt | Nt | 157 |
|  | 4-23 | 4 (0%) | Nt | 65 (0%) | 34 (0%) | Nt | Nt | 103 |
|  |  |  |  |  |  |  |  |  |
|  |  |  |  |  |  |  |  |  |
| pB∆ | 3-03 | 2 (0%) | 1 (100%) | 19 (21%) | 81 (54%) | Nt | Nt | 103 |
|  | 3-33 | 24 (100%) | 9 (100%) | 9 (100%) | Nt | 19 (100%) | 20 (100%) | 81 |
|  | 3-39 | 59 (90%) | 27 (81%) | 124 (97%) | Nt | Nt | 25 (84%) | 235 |
|  | 3-46 | 53 (66%) | 22 (36%) | 57 (46%) | Nt | Nt | 20 (85%) | 152 |
|  | 3-57 | Nt | 320 (98%) | Nt | Nt | Nt | Nt | 320 |
|  | 3-24 | 14 (0%) | Nt | Nt | Nt | Nt | Nt | 14 |
|  | 3-34 | 9 (0%) | Nt | Nt | 116 (0%) | 22 (0%) | Nt | 147 |
|  | 3-47 | 2 (0%) | 34 (0%) | 56 (0%) | 11 (0%) | 44 (0%) | Nt | 147 |
|  | 3-53 | 9 (0%) | 33 (0%) | Nt | Nt | Nt | Nt | 42 |

^a^ Crossing scheme is shown in Figure S1. Nt – not tested
