# Supplementary material for: Specific Tandem Repeats Are Sufficient for Paramutation-Induced Trans-Generational Silencing
Source: PLoS Genet. 2013 Oct 17;9(10):e1003773. doi: 10.1371/journal.pgen.1003773 (PMC3798267; doi:10.1371/journal.pgen.1003773)
Supplement: Table S2 — Frequency of transgene-induced B-I silencing after propagation with a neutral b1 allele. (DOCX) [file pgen.1003773.s010.docx]

**Table S2. Frequency of Transgene-Induced *B-I* Silencing after Propagation with a Neutral *b1* Allele ^a^.**

| Construct | Transgenic event | *TG/-* plants assayed | | Average Frequency of light *B’^#^/ b-N;* |
| --- | --- | --- | --- | --- |
|  |  | Total plants | Light plants | *TG/-* plants (%)^b^ |
| pB | 4-07 | 6 | 1 | 17% |
|  | 4-27 | 143 | 70 | 49% |
|  | 4-36 | 5 | 1 | 20% |
|  | 4-43 | 89 | 20 | 22% |
|  | 4-03 | 83 | 0 | 0% |
|  | 4-06 | 180 | 0 | 0% |
|  | 4-10 | 52 | 0 | 0% |
|  | 4-12 | 147 | 0 | 0% |
|  | 4-14 | 157 | 0 | 0% |
|  | 4-23 | 103 | 0 | 0% |
| Total |  | **965** | **92** |  |
| pB∆ | 3-03 | 103 | 49 | 48% |
|  | 3-33 | 81 | 81 | 100% |
|  | 3-39 | 235 | 216 | 92% |
|  | 3-46 | 152 | 86 | 56% |
|  | 3-57 | 320 | 315 | 98% |
|  | 3-24 | 14 | 0 | 0% |
|  | 3-34 | 147 | 0 | 0% |
|  | 3-47 | 147 | 0 | 0% |
|  | 3-53 | 42 | 0 | 0% |
| Total |  | **1241** | **747** |  |
| pFA | 60-04 | 8 | 8 | 100% |
|  | 60-05 | 12 | 12 | 100% |
|  | 60-11 | 7 | 7 | 100% |
|  | 60-18 | 35 | 35 | 100% |
| Total |  | **62** | **62** |  |
| pFB | 61-03 | 8 | 0 | 0% |
|  | 61-04 | 112 | 0 | 0% |
|  | 61-06 | 28 | 0 | 0% |
|  | 61-07 | 35 | 0 | 0% |
|  | 61-10 | 26 | 0 | 0% |
|  | 61-13 | 29 | 0 | 0% |
|  | 61-28 | 4 | 0 | 0% |
| Total | 61-29 | 5  **247** | 0  **0** | 0% |

^a^ Crossing scheme and summary for the data are shown in Figure S1 and Figure 2.

^b^ The frequency of light plants was calculated by dividing the total number of light transgenic plants over the total number of transgenic plants tested for a given transgenic event.
