# Supplementary material for: Specific Tandem Repeats Are Sufficient for Paramutation-Induced Trans-Generational Silencing
Source: PLoS Genet. 2013 Oct 17;9(10):e1003773. doi: 10.1371/journal.pgen.1003773 (PMC3798267; doi:10.1371/journal.pgen.1003773)
Supplement: Table S3 — DNA blot analysis of the Arabidopsis transgenes. (DOCX) [file pgen.1003773.s011.docx]

**Table S3. DNA Blot Analysis of the Arabidopsis Transgenes^a^**

| Transgenic construct | Number of transgenic events tested | Events with truncated copies only | Events with intact copies only | Events with intact and truncated copies |
| --- | --- | --- | --- | --- |
| pEN-MS1 | 12 | 1 | 6 | 5 |
| pEN-MS2 | 2 |  | 2 |  |
| pEN-MS3 | 15 | 1 | 10 | 4 |
| pEN-MS4 | 7 | 1 | 6 |  |
| pEN-MS5 | 3 |  | 2 | 1 |
| pEN-MS6 | 6 |  | 6 |  |

**^a^** The intactness of the transgenes was checked by DNA blot analysis using enzymes cutting on both sides of the transgenes (*Bam*HI for pEN-MS2 and pEN-MS6, and *Not*I for all other transgenes). The blots were hybridized with the *b1* repeat and luciferase probes.
