# Supplementary material for: Specific Tandem Repeats Are Sufficient for Paramutation-Induced Trans-Generational Silencing
Source: PLoS Genet. 2013 Oct 17;9(10):e1003773. doi: 10.1371/journal.pgen.1003773 (PMC3798267; doi:10.1371/journal.pgen.1003773)
Supplement: Table S4 — Analysis of effect of b1 repeats on expression of GFP reporter gene in Arabidopsis mutants. (DOCX) [file pgen.1003773.s012.docx]

**Table S4. Analysis of Effect of *b1* Repeats on Expression of GFP Reporter Gene in *Arabidopsis* Mutants**

|  | Transgenic events with GFP expression^a^/ Number of  transgenic events examined | | | |
| --- | --- | --- | --- | --- |
| Construct | *drm1 drm2*  mutant | | *rdr2* mutant | *rdr6* mutant |
| pRB1 | 1^b^/34 |  | |  |
| pEN-MS42 | 0/36 | 0/20 | | 0/20 |
| pEN-MS41 | 0/40 | 0/20 | | 0/20 |
| pEN-MS40 | 0/4 |  | |  |
| pRB7 (35S::GFP control) | 29/29 |  | |  |

^a^ GFP expression was assayed using a Leica MZ FLII stereo microscope.

^b^ One pRB1 transgenic event displayed GFP florescence. DNA blot analysis however showed that the transgene in this event lacked the *b1* repeats. This suggests that the GFP florescence was due to the genomic integration site.
